# Deciphering the Molecular Profile of Plaques, Memory Decline and Neuron Loss in Two Mouse Models for Alzheimer’s Disease by Deep Sequencing

**DOI:** 10.3389/fnagi.2014.00075

**Published:** 2014-04-16

**Authors:** Yvonne Bouter, Tim Kacprowski, Robert Weissmann, Katharina Dietrich, Henning Borgers, Andreas Brauß, Christian Sperling, Oliver Wirths, Mario Albrecht, Lars R. Jensen, Andreas W. Kuss, Thomas A. Bayer

**Affiliations:** ^1^Division of Molecular Psychiatry, Georg-August-University Goettingen, University Medicine Goettingen, Goettingen, Germany; ^2^Department of Bioinformatics, Institute of Biometrics and Medical Informatics, University Medicine Greifswald, Greifswald, Germany; ^3^Department of Functional Genomics, Interfaculty Institute for Genetics and Functional Genomics, University Medicine Greifswald, Greifswald, Germany; ^4^Human Molecular Genetics, Department for Human Genetics of the Institute for Genetics and Functional Genomics, Institute for Human Genetics, University Medicine Greifswald, Ernst-Moritz-Arndt University Greifswald, Greifswald, Germany; ^5^Institute for Knowledge Discovery, Graz University of Technology, Graz, Austria

**Keywords:** fear conditioning, spatial reference memory, transcriptome, 5xFAD, Tg4–42, N-truncated abeta, Morris water maze, deep sequencing

## Abstract

One of the central research questions on the etiology of Alzheimer’s disease (AD) is the elucidation of the molecular signatures triggered by the amyloid cascade of pathological events. Next-generation sequencing allows the identification of genes involved in disease processes in an unbiased manner. We have combined this technique with the analysis of two AD mouse models: (1) The 5XFAD model develops early plaque formation, intraneuronal Aβ aggregation, neuron loss, and behavioral deficits. (2) The Tg4–42 model expresses N-truncated Aβ_4–42_ and develops neuron loss and behavioral deficits albeit without plaque formation. Our results show that learning and memory deficits in the Morris water maze and fear conditioning tasks in Tg4–42 mice at 12 months of age are similar to the deficits in 5XFAD animals. This suggested that comparative gene expression analysis between the models would allow the dissection of plaque-related and -unrelated disease relevant factors. Using deep sequencing differentially expressed genes (DEGs) were identified and subsequently verified by quantitative PCR. Nineteen DEGs were identified in pre-symptomatic young 5XFAD mice, and none in young Tg4–42 mice. In the aged cohort, 131 DEGs were found in 5XFAD and 56 DEGs in Tg4–42 mice. Many of the DEGs specific to the 5XFAD model belong to neuroinflammatory processes typically associated with plaques. Interestingly, 36 DEGs were identified in both mouse models indicating common disease pathways associated with behavioral deficits and neuron loss.

## Introduction

Alzheimer disease (AD) is the most common form of dementia in the aging population accounting for 60–80% of the cases. The disease is a progressive neurodegenerative disorder characterized by the presence of extracellular amyloid plaques composed of amyloid-β (Aβ) surrounded by dystrophic neurites and neurofibrillary tangles (NFT) (Alzheimer’s Association, 2012). Further pathological hallmarks of the disease include inflammatory processes, synaptic and neuronal loss, cerebral atrophy, and cerebral amyloid angiopathy (CAA) (Wirths and Bayer, 2012). The complex progression of neurodegeneration in AD patients results in memory impairment and decline in other cognitive abilities often combined with non-cognitive symptoms like mood- and personality changes (Alzheimer’s Association, 2012).

The discovery that certain early-onset familial forms of AD may be caused by an enhanced level of Aβ peptides led to the hypothesis that amyloidogenic Aβ is closely involved in the AD pathogenic process (Selkoe, 1998). The “amyloid hypothesis” that was proposed more than two decades ago claims that extracellular Aβ is the major elicitor of the disease (Hardy and Allsop, 1991). However, while the insoluble fibrillar aggregates of amyloid-β are the main neuropathological hallmark of AD, the plaque load correlates poorly with brain dysfunction and cognitive impairment in AD patients (Price and Morris, 1999; Lesné et al., 2013) or in AD transgenic mouse models (Moechars et al., 1999; Schmitz et al., 2004). In contrast, recent studies indicate that soluble Aβ levels, including soluble oligomers, correlate much better with key features of AD (McLean et al., 1999; Näslund et al., 2000; Selkoe, 2011).

There is increasing evidence that AD is primarily initiated by soluble oligomeric species derived from full-length Aβ_1–42_ (Haass and Selkoe, 2007; Haupt et al., 2012). In addition to soluble oligomers, β-sheet containing amyloid fibrils are also highly toxic forms of Aβ (Klein, 2002). Numerous variants of Aβ_1–42_ oligomers including dimers, trimers, and tetramers have been introduced and are currently discussed as major factors in AD (Roychaudhuri et al., 2009; Benilova et al., 2012). The “modified amyloid hypothesis” now suggests that intraneuronal Aβ accumulation precedes the formation of extracellular plaques and other pathological events in the brains of AD patients (Wirths et al., 2004).

Next to the numerous variants of Aβ_1–42_ oligomers there is substantial evidence that N-terminal truncated peptides play a key role in AD (Jawhar et al., 2011). Besides Aβ peptides starting with an aspartate at position 1, a variety of different N-truncated Aβ peptides have been identified in AD brains. Ragged Aβ peptides, including a major species beginning with phenylalanine at position 4 of Aβ (Aβ_4–42_), have been reported as early as 1985 by Masters et al. (1985).

Only a subgroup of patient families displays the early-onset familial form of AD that is caused by rare single mutations in either the amyloid-protein-precursor (APP) or the presenilin-1 (PSEN-1) and presenilin 2 (PSEN-2) genes. The vast majority of AD patients displays no known mutations and suffers from the sporadic late-onset form of AD (Blennow et al., 2006). To date, the apolipoprotein E (ApoE) ε4 allele is the only known genetic risk factor for sporadic AD (Blennow et al., 2006; Selwood et al., 2009). A variety of additional genetic loci have been proposed to be involved with late-onset AD (Bertram and Tanzi, 2001).

Technical approaches using transcriptome microarray analyses were performed over the last years to identify genes that are differentially expressed and therefore may be involved in the pathophysiology of AD (George et al., 2010).

The recent developments in next-generation sequencing (deep sequencing) offer a more comprehensive and most of all unbiased approach for transcriptome analysis. Multiple studies already indicate that next-generation sequencing is more useful and particularly suitable to investigate the pathogenesis of complex neurodegenerative diseases like AD (Twine et al., 2011). For example, Sultan et al. (2008) claimed that deep sequencing of non-ribosomal RNA (RNA-Seq) could detect up to 25% more genes compared to microarrays analyses.

In the present study, we performed a comparative gene expression analysis of brain tissue of two different mouse models for AD using next-generation sequencing. We compared the well-established, plaque-developing 5XFAD mouse model (Oakley et al., 2006) with the Tg4–42 mouse model that solely expresses Aβ_4–42_ without extracellular plaque deposition (Bouter et al., 2013). The aim of this study was to elucidate the similarities and distinctions in expression profiles of these two mouse models that display similar memory deficits.

## Materials and Methods

### Transgenic mice

In this study, we used the transgenic mouse lines Tg4–42 and 5XFAD. The generation of Tg4–42 has been recently described by our lab (Bouter et al., 2013). Tg4–42 mice express human Aβ_4–42_ fused to the murine TRH signal peptide under the control of the neuronal Thy-1 promoter.

5XFAD mice over-express the 695 amino acids isoform of the human amyloid precursor protein (APP695) carrying the Swedish, London, and Florida mutations under the control of the murine Thy-1 promoter. In addition, human presenilin-1 (PSEN-1) carrying the M146L/L286V mutations is expressed also under the control of the murine Thy-1 promoter (Oakley et al., 2006). 5XFAD mice used in the current study were backcrossed for more than eight generations to C57Bl/6J wildtype mice (Jackson Laboratories, Bar Harbor, ME, USA) to obtain an incipient congenic line on a C57Bl/6J genetic background (Jawhar et al., 2010). Young (3–6 months) and aged (12 months) Tg4–42, 5XFAD mice, and wildtype (WT, C57BL/6J) controls were tested. In the current study, only female mice were used. Wildtype littermate control mice served as age-matched control animals. All animals were handled according to the German guidelines for animal care. All efforts were made to minimize suffering and the number of animals used for this study.

### Spatial reference memory by Morris water maze

Spatial reference memory in Tg4–42 and 5XFAD mice was evaluated using the Morris water maze (Morris, 1984) as described previously (Bouter et al., 2013). In brief, mice learn to use spatial cues to locate a hidden platform in a circular pool filled with opaque water. The pool was divided into four virtual quadrants that were defined based on their spatial relationship to the platform: left, right, opposite, and target quadrant, which contains the goal platform. ANY-Maze video tracking software (Stoelting Co., Wood Dale, IL, USA) was used to record escape latency, swimming speed, and quadrant preference.

Young and aged Tg4–42, 5XFAD mice, and wildtype (WT, C57BL/6J) controls were tested (*n* = 8–11 mice per group).

The experiment began with 3 days of cued training during which the platform was marked with a triangular flag. Both the location of the platform and the position where mice were introduced into the pool changed between trials. Each mouse received four training trials per day with an average inter-trial interval of 15 min.

Twenty-four hours after the last day of cued training, mice performed 5 days of acquisition training. For this part of testing, the flag was removed from the platform. In addition to the distal cues existing in the room, proximal visual cues were attached to the outside of the pool. The platform location remained stationary for each mouse throughout training. Trials were conducted as during the cued training phase.

Twenty-four hours after the last acquisition trial, a probe test was performed to assess spatial reference memory. The platform was removed from the pool, and mice were introduced into the water from a novel entry point. Mice were then allowed to swim freely for 1 min while their swimming path was recorded. After the probe trial, the mice were sacrificed.

### Contextual and tone fear conditioning

Twelve-month-old Tg4–42, 5XFAD, and WT mice were subjected to contextual fear conditioning (CFC) and tone fear conditioning (TFC) (*n* = 11–13). A 3-day delay fear conditioning protocol was used to assess conditional learning and memory. According to this protocol, the *conditioned stimulus* (CS) is presented and overlapped by the presentation of the *unconditioned stimulus* (US) (Ohno, 2009).

The experiments were performed using a standard conditioning chamber (17 cm × 17 cm × 26 cm) with a stainless steel grid floor connected to a shock generator (Ugo Basile Sound and Shocker Generator, Comerio, Italy). The walls were covered with black and white checkered paper (CS). The chamber was housed in a soundproof isolation cubicle. A digital camera and an additional light source were attached to the ceiling of the cubicle. ANY-Maze video tracking software (Stoelting Co., Wood Dale, IL, USA) was used to record freezing behavior of animals.

On day one, mice were placed in the conditioning chamber and allowed to explore the box for 150 s. After the habituation period, a tone (2000 Hz, 80 dB; CS) was presented for 30 s that simultaneously ended with a 2 s foot-shock (0.7 mA, US). Mice were allowed to recover after the foot-shock for an additional 30 s before being returned to their home cage. Baseline freezing was recorded before the presentation of the tone.

Twenty-four hours after the training mice were placed back in the familiar fear conditioning chamber, but in the absence of tones and foot-shocks. Freezing behavior was measured for 210 s to test contextual memory retrieval.

For the tone fear retrieval trial on day 3, mice were placed for 3 min in an altered conditioning chamber with white walls, a covered floor, and an acetic acid scent. After 150 s baseline recording, a tone similar to the one used during the fear conditioning trial was presented for 30 s. The freezing behavior before and during the CS tone was measured. Mice were sacrificed after the tone trial.

### Statistical analysis of behavior experiments

Differences between groups were tested with unpaired *t*-test, one-way analysis of variance (ANOVA) followed by Bonferroni multiple comparisons or repeated measures ANOVA followed by Bonferroni multiple comparisons as indicated. All data are given as means ± standard error of the mean (SEM). Significance levels are given as follows: ***p < 0.001; **p < 0.01; *p < 0.05. All statistics were calculated using STATISTICA version 10.0 for Windows (StatSoft, Tulsa, OK, USA) and GraphPad Prism version 5.04 for Windows (GraphPad Software, San Diego, CA, USA).

### Tissue harvesting

Mice were sacrificed via CO_2_ anesthetization followed by cervical dislocation. Brain hemispheres were carefully dissected (olfactory bulbs and cerebellum was removed), frozen on dry-ice and stored at −80°C for subsequent use.

### RNA expression profiling

Expression profiling for young and aged Tg4–42, 5XFAD, and WT mice was performed by next-generation sequencing on a SOLiD 5500xl Genetic Analyzer (Life Technologies, Carlsbad, CA, USA). RNA was extracted from mouse brain hemispheres as follows. The tissue was homogenized using a Polytron (VWR) device and then treated with TRIzol (Life Technologies, Carlsbad, CA, USA). Next, 5 μg of each total RNA sample were spiked with ERCC spike-in control mixes (Life Technologies, Carlsbad, CA, USA) before removal of the rRNA by use of a RiboZero Kit (Epicentre, Madison, WI, USA). The RNA was prepared for sequencing following the protocol provided by the manufacturer of the sequencer. In brief, the rRNA depleted RNA was fragmented by chemical hydrolysis, phosphorylated, and purified. Adaptors were ligated to the RNA fragments, which subsequently were reverse transcribed into cDNA. The cDNA was purified and size-selected using two rounds of Agencourt AMPure XP bead purification (Beckman Coulters Genomics, Danvers, MA, USA) and released from the beads. The sample was amplified by 12 PCR cycles in the presence of primers that contained unique sequences (barcoding). The size distribution and concentration of the fragments were determined with an Agilent 2100 Bioanalyzer and the corresponding chemicals (Agilent Technologies, Santa Clara, CA, USA).

The cDNA fragments were pooled in equimolar amounts and diluted to 76 pg/μL corresponding to a concentration of 500 pM. Fifty microliters of this dilution was mixed with a freshly prepared oil emulsion, P1 and P2 reagents, and P1 beads in a SOLiD EZ Bead Emulsifier prepared according to the E80 scale protocol (Life Technologies, Carlsbad, CA, USA). The emulsion PCR was carried out in a SOLiD EZ Bead Amplifier (Life Technologies, Carlsbad, CA, USA) using the E80 setting. To enrich for the beads that carried amplified template DNA, the beads were purified on a SOLiD EZ Bead Enricher using the recommended chemicals and software (Life Technologies, Carlsbad, CA, USA). The purified beads were loaded onto a SOLiD 6-lane Flowchip and incubated upside down for 1 h at 37°C. The Flowchip was positioned in the 5500xl SOLiD System and the DNA was sequenced using the settings and recommended chemicals for sequencing 75 nucleotides in the forward direction and 35 nucleotides in the reverse direction (Life Technologies, Carlsbad, CA, USA).

Sequence reads were mapped to the mouse genome reference sequence mm10^1^ using the workflow “whole.transcriptome.pe” LifeScope-v2.5.1-r0 (Life Technologies, Carlsbad, CA, USA). Reads mapping to RefSeq coding exons (accessed 2012-06-27)^2^ and matching the coding strand were considered as coding RNAs. All other mapping reads were considered non-coding.

### Differential expression analysis

RNA-Seq read data were normalized within and between lanes for GC-content using EDASeq’s full-quantile normalization (Risso et al., 2011). The differential expression analysis was done with DESeq (Anders and Huber, 2010). All samples were treated as replicates of a single condition for the estimation of the dispersion. Only the fitted dispersion values were used in the following analyses. The significance of differential expression was determined by the Benjamini–Hochberg corrected *p*-values of the negative binomial test between two conditions. The threshold for significance was set to *p* = 0.05. The following conditions were compared: young WT vs. young Tg4–42, young WT vs. young 5XFAD, aged WT vs. aged Tg4–42, and aged WT vs. aged 5XFAD. Genes with more than 200 reads were successfully verified by real-time quantitative PCR (qRT-PCR) and are listed in the results part. Genes with an expression level lower than 200 reads are not shown.

### Real-time quantitative PCR confirmation

RNA was isolated from female young and aged 5XFAD mice, aged Tg4–42, and aged-matched WT mice (*n* = 5 each) as described previously (Hillmann et al., 2012). Briefly, frozen right brain hemispheres were homogenized with 10 strokes of a R50D homogenizer (CAT) set at 800 rpm in 1.5 mL ice-cold Trifast^®^ (Peqlab, Erlangen, Germany). Three hundred microliters chloroform (Merck) was added to each sample. After 10 min incubation, the samples were centrifuged at 12000 × *g* for 15 min at 4°C to separate the RNA. The upper RNA-containing aqueous phase was transferred into a new tube, vigorously mixed with 500 μL Isopropanol, and incubated for 20 min on ice. After centrifugation at 12000 × *g* for 10 min at 4°C, the supernatant was discarded. RNA pellets were washed twice with 500 μL 75% Ethanol. After the pellet was air-dried, the RNA was dissolve in 30 μL of RNAse free water. RNA was stored at −80°C until further use. RNA purity and yields were determined by a Biophotometer (Eppendorf, Hamburg, Germany).

Total RNA (1 μg) was subjected to reverse transcription to synthesize cDNA using the First-Strand cDNA Synthesis Kit (Fermentas, St. Leon-Rot, Germany) according to the manufacturer’s instructions. Prior to reverse transcription, RNA was subjected to digestion by DNase using a DNase Digestion Kit (Fermentas, St. Leon-Rot, Germany). Generated cDNA was diluted 1:10 in ddH_2_O and used as the sample template for qRT-PCR. The obtained cDNA was stored at −20°C until use.

Quantitative PCR was used to validate the results obtained from the deep sequencing analysis. Several genes were selected for both transgenic mouse lines and time points. Primers were purchased from Eurofins (Ebersberg, Germany) as intron-spanning validated primer pairs. The diluted first-strand cDNA was used for qRT-PCR using the SYBR green based DyNAmo Flash SYBR Green qPCR Kit (Thermo Fischer Scientific, Waltham, MA, USA) containing ROX as an internal reference dye. Samples were normalized to the housekeeping gene β-Actin.

Analysis of brain transgene expression in 5XFAD, Tg4–42, and WT animals was performed in the MX3000P Real-Time Cycler (Stratagene, Santa Clara, CA, USA) and data collected using the MxPro Mx3000P software (Stratagene, Santa Clara, CA, USA). Statistical analysis of quantitative RT-PCR measurements was done using the Relative Expression Software Tool V1.9.6 (REST, Qiagen, Hilden, Germany) (Pfaffl et al., 2002). The expression ratio results of the studied transcripts were tested for significance by Pair Wise Fixed Reallocation Randomization Test. ***p < 0.001; **p  < 0.01; *p < 0.05.

### Annotation analysis

In order to gain insight in the biological function and to understand the biological significance of differentially expressed genes (DEGs), the functional annotation of DEGs was obtained using Source^3^, GeneCards^4^, Wiki-Pi^5^, and Mouse Genome Informatics^6^.

## Results

### Tg4–42 and 5XFAD mice display spatial memory deficits

Spatial reference memory was assessed in Tg4–42, 5XFAD, and WT mice using the Morris water maze. First, mice performed cued training with a marked platform to familiarize with the pool and to rule out effects from possible motor or sensory deficits. WT, Tg4–42, and 5XFAD mice showed progressively decreased escape latencies at all ages tested and no differences in swimming speed (data not shown). The cued training revealed that all mice had an intact vision and appropriate motor abilities to swim.

Twenty-four hours after the cued training, mice were subjected to acquisition training in order to test their learning abilities to find the location of a submerged platform using distal and proximal cues.

**Figure 1:**
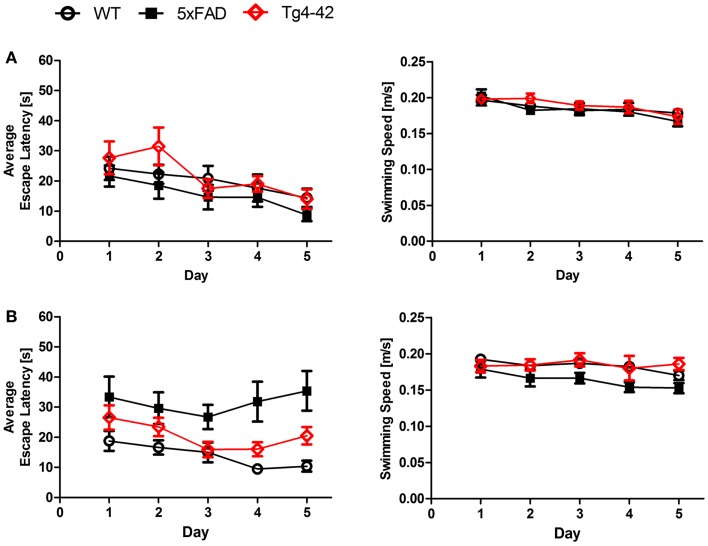
**Spatial learning deficits in aged Tg4–42 and 5XFAD shown in the acquisition training of the Morris water maze**. Female **(A)** young and **(B)** aged Tg4–42 mice, 5XFAD mice, and WT littermate controls were tested (*n* = 8–11). Animals tested underwent acquisition training to learn to use distal and proximal cues to navigate a direct path to a hidden platform. Escape latencies of young mice **(A)** decreased progressively over 5 days of training regardless of the genotype. Furthermore, aged WT mice **(B)** showed a progressive improvement in the escape latency over time. The escape latencies for aged Tg4–42 and 5XFAD did not improve over the 5 days of training. Swimming speed was not affected in all mice tested. Escape latency and swimming speed: repeated measures ANOVA followed by Bonferroni multiple comparisons. *m* age in months.

We found a significant main effect of *genotype* for escape latencies (Repeated measures ANOVA, *F* = 3.4097; *p* = 0.04). Young Tg4–42, 5XFAD, and WT mice showed a significant decrease in the escape latencies to reach the hidden platform (Figure 1A, Repeated measures ANOVA, escape latency: *p* = 0.000011). Moreover, aged WT animals showed a significant decrease in the escape latencies while the escape latencies for aged Tg4–42 and 5XFAD did not improve over the 5 days of training (Figure 1B, Repeated measures ANOVA, escape latency: *p* = 0.001).

In contrast, the swimming speed across the 5 days of acquisition training showed no significant difference irrespective of genotype and age (Figures 1A,B, Repeated measures ANOVA, *p* = 0.0566).

Young Tg4–42 and 5XFAD animals performed superior to older animals while this difference was not due to differences in swimming velocity due to age-related motor deficits. These results suggest that spatial learning is impaired in aged Tg4–42 and 5XFAD mice.

**Figure 2:**
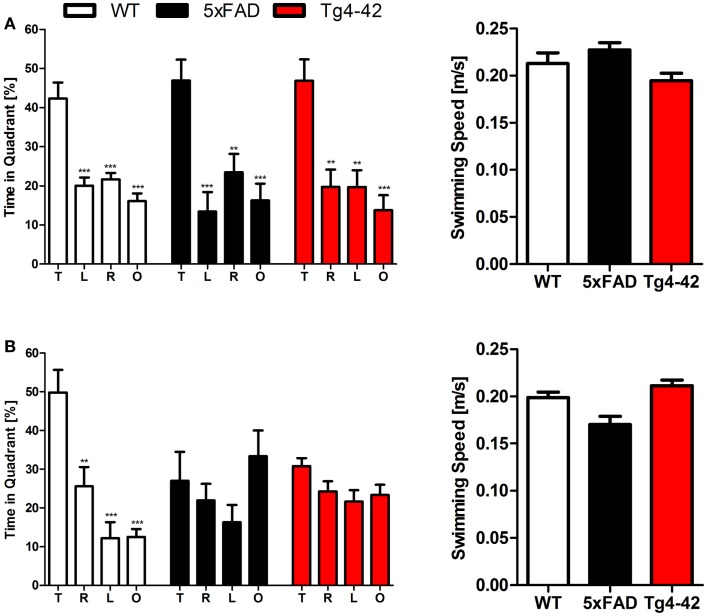
**Spatial reference memory deficits in aged Tg4–42 and 5XFAD mice shown in the probe trial of the Morris water maze**. Female young and aged Tg4–42 mice, 5XFAD mice, and WT littermate controls were tested (*n* = 8–11). The probe trial was given at the end of learning phase (acquisition training) to assess spatial reference memory. Quadrant preference and swimming speed for the first 30 s of the probe trial were analyzed. **(A)** Young Tg4–42, 5XFAD, and WT mice showed no impairment in spatial reference memory. All groups spent a significant greater percentage of time in the target quadrant (WT: *p* < 0.001 T vs. all other quadrants; 5XFAD: *p* < 0.001 T vs. L and O, *p* < 0.01 T vs. R; Tg4–42: *p* < 0.001 T vs. O, *p* < 0.01 T vs. R and L). The swimming speed did not differ between the groups. **(B)** Probe trial revealed a significant reduced learning behavior for aged Tg4–42 and 5XFAD mice as they showed no preference for the target quadrant. WT mice have no learning deficits at this age (WT: *p* < 0.001 T vs. L and O, *p* < 0.01 T vs. R). Swimming speed did not differ between the groups. T, target quadrant; L, left quadrant; R, right quadrant; O, opposite quadrant. Quadrant preference and swimming speed; One-way analysis of variance (ANOVA) followed by Bonferroni multiple comparisons. ***p < 0.001; **p < 0.01.

Twenty-four hours after the last acquisition trial, a probe trial was given to assess spatial reference memory. Young Tg4–42, 5XFAD, and WT mice displayed a significant preference for the target quadrant, as indicated by the percentage time spent in different quadrants of the pool (Figure 2A, One-way ANOVA, WT: *p* < 0.0001, df = 3; *p* < 0.001 target vs. all other quadrants; 5XFAD: *p* < 0.0001, df = 3; *p* < 0.001 target vs. left and opposite quadrant, *p* < 0.01 target vs. right quadrant; Tg4–42: *p* < 0.0001, df = 3; *p* < 0.001 target vs. opposite quadrant, *p* < 0.01 target vs. right quadrant).

No quadrant preference was found for aged Tg4–42 and 5XFAD mice, while WT mice still demonstrated significant preference for the target quadrant at that time point (Figure 2B, One-way ANOVA, WT: *p* < 0.0001, df = 3; *p* < 0.001 target vs. left and opposite quadrant, *p* < 0.01 target vs. right quadrant). Swimming speed between the groups did not differ during the probe trial. The absence of a preference for the target quadrant as compared to the remaining quadrants during the probe trial demonstrates that aged Tg4–42 and 5XFAD mice display a robust deficit in spatial reference memory.

In summary, the results of the acquisition phase and the probe trial suggest that aged Tg4–42 and 5XFAD mice display an impaired spatial and spatial reference memory.

### Tg4–42 and 5XFAD mice exhibit decreased contextual learning

**Figure 3:**
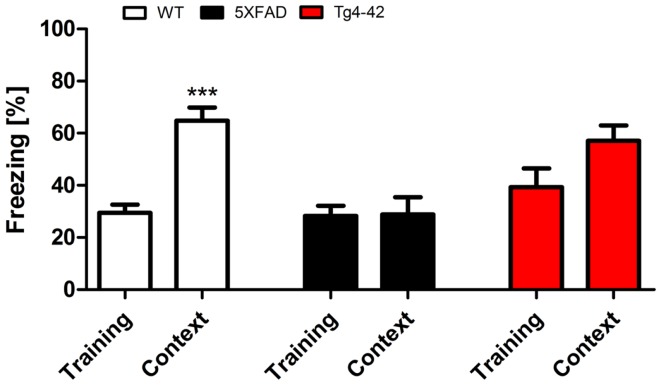
**Impaired contextual conditioning in Tg4–42 and 5XFAD mice**. Aged 5XFAD, Tg4–42, and WT mice were trained with a CS/US pairing for contextual fear conditioning (*n* = 11–13). Mice were reintroduced to the original training context (CS) 24 h post training and tested for contextual memory. Levels of freezing during the re-exposure were not different from the training trial for 5XFAD and Tg4–42. In contrast, WT mice showed a significant increase on freezing response to the context. CS = conditioned stimulus. Freezing: unpaired *t*-test;***p < 0.001.

During the initial training sessions involving tone-foot-shock pairing (CS/US), 12-month-old Tg4–42, 5XFAD, and WT mice exhibited comparable degrees of freezing (Figure 3). When mice were tested for context fear conditioning 24 h after the training trial, Tg4–42 and 5XFAD mice demonstrated no significantly increased freezing behavior in response to the conditioning chamber (Figure 3). In contrast, WT mice displayed significantly increased freezing (One-way ANOVA, *p* < 0.001) as a response to the context.

Tg4–42 and 5XFAD mice jumped and vocalized in response to the electric foot-shock to a similar degree as WT mice, suggesting normal pain perception in these mutant mice. However, transgenic mice were not able to attribute the pain of the foot-shock during the training trial to the context. Therefore, Tg4–42 and 5XFAD show impaired contextual learning.

### 5XFAD mice show impaired tone learning

**Figure 4:**
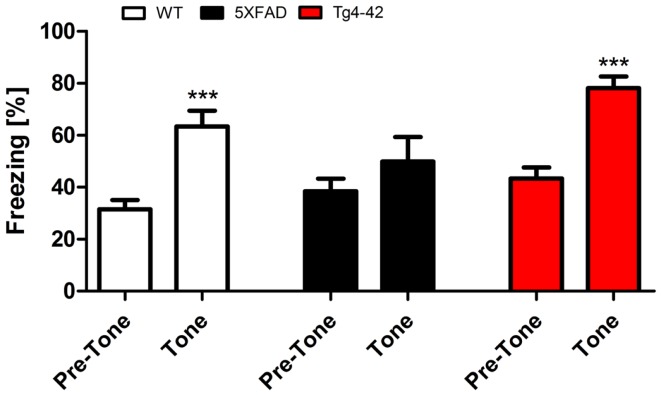
**Impaired tone conditioning in 5XFAD mice**. Aged 5XFAD, Tg4–42, and WT mice at 12 months of age were trained with a CS/US pairing for tone fear conditioning (*n* = 11–13). Mice were placed in an altered fear conditioning chamber 48 h post training and tested for freezing during tone presentation (CS). WT and Tg4–42 mice shock froze significantly more during tone presentation compared to the training trial. In contrast, 5XFAD mice did not associate the tone with the received foot-shock as freezing was not significantly different between the training and the tone trial. CS, conditioned stimulus; US, unconditioned stimulus. Freezing: unpaired *t*-test;**p < 0.001.

Twenty-four hours after the context testing (48 h after training), the same mice were tested for conditioned fear of a tone. Therefore, mice were reintroduced to the altered fear conditioning chamber. When the tone was presented without the foot-shock, both Tg4–42 and WT mice exhibited similar freezing responses (Figure 4). In both mouse lines, freezing increased significantly compared to the pre-tone period (One-way ANOVA, WT, and Tg4–42: *p* < 0.001). However, 5XFAD mice demonstrated substantially less freezing behavior in response to the tone. 5XFAD mice did not associate the tone with the previously received foot-shock as freezing was not significantly different between the training and the tone trial.

These results indicate that Tg4–42 mice exhibit a selective impairment of contextual fear learning (see previous sections), while their tone learning ability remains intact. 5XFAD mice on the other hand demonstrate both impaired contextual and tone fear learning.

### Deep sequencing of mouse brains

In total, deep sequencing identified 15,711,910 and 16,143,760 sequence reads for young and old wildtype mice, respectively. For young wildtype mice, 6,230,197 reads (39.65%) and for old wildtype mice, 5,512,056 reads (34.14%) were mapped to exons. In young 5XFAD mice, the read mapping revealed 8,570,239 (60.28%) of 14,216,258 reads in exonic regions. Out of 18,288,161 reads, 9,163,060 (50.10%) hit exons in old 5XFAD mice. The brain exome of young Tg4–42 mice was covered by 6,342,018 (47.28%) out of 13,414,301 reads. For old Tg4–42 mice, 12,488,206 reads were detected in total, of which 4,976,552 (39.85%) could be mapped to exons. The numbers of exonic reads are summarized in Table 1.

**Table 1:** **Number of exonic reads in brain tissue of wildtype and transgenic mice**.

Genotype	Number of reads in exons
Young WT	6,230,197
Aged WT	5,512,056
Young 5XFAD	8,570,239
Aged 5XFAD	9,163,060
Young Tg4–42	6,342,018
Aged Tg4–42	4,976,552

### Deep sequencing identified over-expressed transgenes

5XFAD mice over-express human amyloid precursor protein (APP695) carrying the Swedish, London, and Florida mutations as well as human presenilin-1 (PSEN-1) carrying the M146L/L286V mutations. Both peptides are expressed under the control of the neuronal Thy-1 promoter (Oakley et al., 2006). As expected, sequence reads pertaining to PSEN-1, APP, and a Thy-1 promoter sequence (Moechars et al., 1996) were over-represented in both young and aged old 5XFAD brains (data not shown) and therefore served as a positive and internal control for RNA-Seq.

In Tg4–42 mice, a Thy-1 promoter sequence (Moechars et al., 1996) was found to be over-expressed in both young and aged mice (data not shown). Again, this was expected as Tg4–42 mice express human Aβ_4–42_ fused to the murine TRH signal peptide under the control of the neuronal Thy-1 promoter (Bouter et al., 2013).

### Gene expression in young Tg4–42 and 5XFAD mice

**Figure 5:**
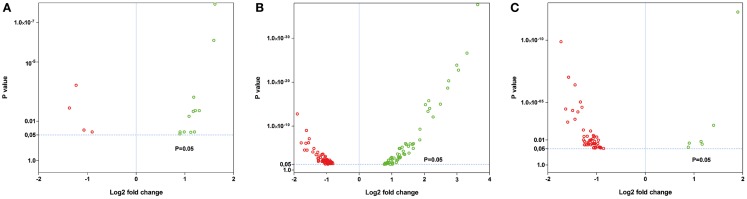
**Volcano plots of the significant gene expression changes in Tg4–42 and 5XFAD mice**. Fold changes in gene expression of **(A)** young 5XFAD, **(B)** aged 5XFAD, and **(C)** aged Tg4–42 mice. Each dot represents one gene. Dashed lines illustrate statistical significance (*p* = 0.05). Red, down-regulated; green, up-regulated.

Nineteen genes were identified as significantly differentially expressed between young 5XFAD and age-matched WT mice. In order to demonstrate the expression changes, volcano plots were created (Figure 5A). Thirteen genes were up-regulated (Figure 5A, green dots), while six genes were down-regulated (Figure 5A, red dots). DEGs encoded proteins from diverse functional categories, including translation (ribosomal proteins), glycolysis, and ATP-binding, kinases and hydrolases (Table 2). In contrast, no DEGs could be detected in young Tg4–42 mice.

**Table 2:** **List of differentially expressed transcripts in young 5XFAD mice**.

ID	Gene name	Gene description	GO biological process annotation/functions	Log2 fold change	Adjusted *p*-value
MGI:87994	*Aldoa*	Aldolase A, fructose-bisphosphate	Fructose-bisphosphate aldolase activity	1.62	1.16E-08
			Actin binding	
			Cytoskeletal protein binding	
			Tubulin binding	
			Glycolysis	
MGI:2148181	*Snora68*	Small nucleolar RNA, H/ACA box 68	Non-coding RNA	1.60	7.99E-07
			Uridine modifications	
MGI:105110	*Rps2*	Ribosomal protein S2	mRNA binding	1.30	2.92E-03
			Fibroblast growth factor binding	
			Structural constituent of ribosome	
MGI:96412	*Ide*	Insulin-degrading enzyme	Insulysin activity	1.22	2.92E-03
			Metalloendopeptidase activity	
			Protein homodimerization activity	
			Hydrolase Activity	
			Beta-amyloid binding	
			Glycoprotein binding	
			ATP-binding	
			Zinc ion binding	
			Ubiquitin binding	
MGI:1353472	*Rpl7a*	Ribosomal protein L7a	RNA binding	1.20	3.51E-02
			Structural constituent of ribosome	
MGI:98865	*Ttr*	Transthyretin	Hormone activity	1.18	6.09E-04
			Protein heterodimerization activity	
			Retinol binding	
MGI:1340062	*Sgk1*	Serum/glucocorticoid regulated kinase 1	Kinase activity	1.17	3.16E-03
			Potassium/calcium channel regulator activity	
			ATP-binding	
			Response to DNA damage stimulus	
MGI:1278340	*Rpl21*	Ribosomal protein L21	Structural constituent of ribosome	1.12	3.65E-02
			RNA binding	
MGI:108415	*Pafah1b2*	Platelet-activating factor acetylhydrolase, isoform 1b, subunit 2	Hydrolase activity	1.09	5.67E-03
			1-Alkyl-2 acetylglycero-phosphocholine esterase activity	
			Homodimerization activity	
MGI:99845	*Gdi2*	Guanosine diphosphate (GDP) dissociation inhibitor 2	Rab GDP-dissociation inhibitor activity	0.99	3.51E-02
			Rab GTPase activator activity	
MGI:1934664	*Rpph1*	Ribonuclease P RNA component H1	Endoribonuclease activity	0.90	3.51E-02
MGI:97783	*Psap*	Prosaposin	Glycoprotein	0.90	3.51E-02
			Lipid binding	
			Enzyme activator activity	
MGI:97591	*Pkm*	Pyruvate kinase, muscle	Magnesium ion binding	0.90	4.41E-02
			ATP-binding	
			Potassium ion binding	
			Pyruvate kinase activity	
MGI:108391	*Kif1a*	Kinesin family member 1A	ATP-binding	−0.90	3.51E-02
			Phospholipid binding	
			Motor activity	
			Axonal neuropathies	
MGI:88106	*Atp1a2*	ATPase, Na+/K+ transporting, alpha 2 polypeptide	Sodium:potassium-exchanging ATPase activity	−0.90	3.51E-02
			ATP-binding	
			Metal ion binding	
			Hydrolase activity	
MGI:1860283	*Ubqln2*	Ubiquilin 2	Protein binding	−1.07	2.82E-02
			Cell death	
MGI:1313261	*Spnb3*	Spectrin beta, non-erythrocytic 2	Phospholipid binding	−1.23	1.50E-04
			Actin binding	
			Structural constituent of cytoskeleton	
MGI:1337000	*Rn45s*	45S pre-ribosomal 5	Non-coding RNA	−1.23	1.50E-04
MGI:104296	*Nova2*	Neuro-oncological ventral antigen 2	RNA binding	−1.37	2.14E-03

### Gene expression in aged Tg4–42 mice

**Figure 6:**
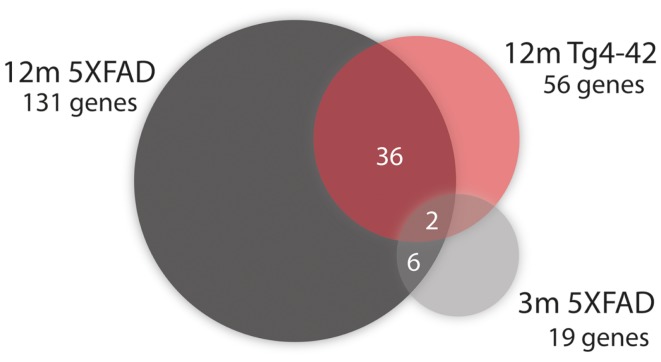
**Venn diagram analysis for significantly regulated genes in Tg4–42 mice compared to 5XFAD mice**. The numbers outside each circle represent the number of genes that were significantly differentially expressed in the respective mouse line (compared to WT mice). The numbers in the spaces of overlapping circles represent the number of genes that were affected in more than one condition.*p* < 0.05.

Fifty-six genes were differentially expressed in aged Tg4–42 mice. Seven genes were up-regulated and 49 down-regulated (Figure 5C). Twenty genes were solely differentially expressed in aged Tg4–42 (Table 3; Figure 6), among these only three genes were found to be up-regulated (*Uqcc2*, *Beta-S*, and *Kif1a*).

**Table 3:** **List of transcripts exclusively differentially expressed in aged Tg4–42**.

ID	Gene name	Gene description	GO biological process annotation/functions	log2 Fold change	Adjusted *p*-value
MGI:1914517	*Uqcc2*	Ubiquinol-cytochrome-*c* reductase complex assembly factor 2	Regulation of insulin secretion	1.17	2.18E-02
			ATP production	
MGI:5474852	*Beta-S*	Hemoglobin, beta adult s chain	Iron ion binding	1.14	1.31E-02
			Oxygen binding	
MGI:108391	*Kif1a*	Kinesin family member 1A	Microtubule motor activity	0.90	1.77E-02
			ATP-binding	
MGI:2153272	*Trrap*	Transformation/transcription domain-associated protein	Phosphotransferase Activity	−0.86	4.74E-02
			Regulation of transcription	
MGI:1194504	*Kcnj10*	ATP-sensitive inward rectifier potassium channel 10	Potassium channel activity	−0.93	3.76E-02
			ATP-binding	
MGI:3039582	*Lmtk3*	Lemur tyrosine kinase 3	Protein tyrosine kinase activity	−0.94	3.98E-02
MGI:1343180	*Vgf*	Nerve growth factor inducible	Neuropeptide hormone activity	−0.95	3.64E-02
			Synaptic plasticity	
			Neurosecretory protein (Jahn et al., 2011 no. 284)	
			Regulation of energy balance (Jahn et al., 2011 no. 284)	
			Important for modulating neuronal activity (Cocco et al., 2010 no. 286)	
MGI:106374	*Zmiz2*	Zinc finger MIZ domain containing protein 2	Zinc ion binding	−0.98	1.90E-02
			Ligand-dependent nuclear receptor transcription coactivator activity	
MGI:1194488	*Slc32a1*	Vesicular inhibitory amino acid transporter solute carrier family 32 (GABA vesicular transporter), member 1	Glycine transporter activity	−1.02	3.76E-02
			Amino acid-polyamine transporter activity	
			Neurotransmitter transport	
MGI:1277171	*Dcx*	Doublecortin	Microtubule binding	−1.03	4.42E-02
			Protein kinase binding	
			Neurogenesis	
MGI:101947	*Hnrnpd*	Heterogeneous nuclear ribonucleoprotein D	Regulation of transcription	−1.04	1.99E-02
			RNA binding and telomeric DNA binding	
MGI:109591	*Nfic*	Nuclear factor I/C	Transcription factor activity	−1.10	1.90E-02
			DNA binding	
MGI:2441726	*BC005537*	cDNA sequence BC005537	Unknown	−1.11	9.46E-03
MGI:2673998	*Arhgap33*	Rho GTPase activating protein 33	Rac GTPase activator activity	−1.12	1.08E-02
			Phosphatidylinositol binding	
MGI:1330828	*Cdk5r2*	Cyclin-dependent kinase 5 activator 2 (p39)	Lipid binding	−1.14	1.04E-02
			Cyclin-dependent protein kinase 5	
			Activator activity	
			Neuron-specific	
MGI:2674092	*Zfp609*	Zinc finger protein 609	Zinc ion binding	−1.17	1.90E-02
MGI:1915454	*2900060*	RIKEN cDNA 2900060B14 gene	Unknown	−1.18	2.43E-02
	*B14Rik*	
MGI:1351334	*Syn3*	Synapsin III	Catalytic activity	−1.25	1.30E-02
			ATP-binding	
MGI:1920907	*Fbrsl1*	Fibrosin-like 1	Unknown	−1.25	2.43E-02
MGI:106589	*Hivep3*	Human immunodeficiency virus type I enhancer binding protein 3	DNA binding	−1.26	5.18E-03
			Zinc ion binding		
			Transcription factor		

The 17 genes that were significantly down-regulated are involved in diverse biological processes including regulation of gene expression, nervous system development, cell communication, metal ion transport, neurogenesis, and regulation of synaptic plasticity.

### Genes similarly expressed in both aged Tg4–42 and 5XFAD mice

Of the 56 DEGs in aged Tg4–42 mice, 36 were also found to be differentially expressed in aged 5XFAD mice (Table 4; Figure 6). Of these 36 genes, four were up-regulated and 32 were down-regulated in aged Tg4–42 and 5XFAD mice and most showed similar expression levels in the two models.

**Table 4:** **List of transcripts differentially expressed in both aged Tg4–42 and 5XFAD mice**.

ID	Gene name	Gene description	GO biological process annotation/functions	log2 Fold change Tg4–42	Adjusted *p*-value Tg4–42	log2 Fold change 5xFAD	Adjusted *p*-value 5xFAD
MGI:103249	*Calm3*	Calmodulin 3	Ion channel binding	1.90	5.49E-13	1.00	1.28E-03
			Calcium ion binding	
			G-protein coupled receptor protein signaling pathway	
MGI:2446216	*Fbxo2*	F-box protein 2	Ubiquitin-protein ligase activity	1.40	6.60E-04	1.24	1.73E-03
			Glycoprotein binding	
			Beta-amyloid binding	
			Carbohydrate binding	
MGI:107671	*Gpm6a*	Neuronal membrane glycoprotein M6-a	Calcium channel activity involved in neuronal differentiation	0.90	1.77E-02	0.90	5.39E-03
			Role in neuronal plasticity	
MGI:95697	*Gfap*	Glial fibrillary acidic protein	Integrin binding	0.88	3.76E-02	3.64	2.36E-42
			Kinase binding	
			Structural constituent of cytoskeleton	
MGI:1860283	*Ubqln2*	Ubiquilin 2	Ubiquitin binding	−0.92	4.35E-02	−1.27	8.71E-05
			Protein modification	
			Proteolysis	
MGI:97495	*Pbx1*	Pre-B-cell leukemia transcription factor 1/pre-B-cell leukemia homeobox 1	Transcription factor activity	−0.92	3.79E-02	−0.86	2.85E-02
			Protein heterodimerization activity involved in the regulation of osteogenesis required for skeletal patterning and programing	
MGI:3647820	*Gm15800*	Predicted gene 15800	Ubiquitin-protein ligase activity	−0.96	1.04E-02	−0.83	1.84E-02
MGI:96669	*Kcnc3*	Potassium voltage-gated channel, shaw-related sub-family, member 3	Voltage-gated potassium channel activity	−0.99	3.47E-02	−1.04	6.53E-03
			Delayed rectifier potassium channel activity	
MGI:96828	*Lrp1*	Low density lipoprotein receptor-related protein 1	Endocytic receptor or receptor activity	−1.00	5.80E-03	−0.83	1.77E-02
			Lipoprotein binding	
			Calcium ion binding	
			Apolipoprotein binding	
			Beta-amyloid clearance	
			Apoptotic cell clearance	
MGI:2183691	*Nav2*	Neuron navigator 2	Heparin binding	−1.04	1.29E-02	−0.89	2.33E-02
			Helicase activity	
			ATP-binding	
			Role in neuronal development	
MGI:1890563	*Wasf1*	WAS protein family, member 1	Actin binding	−1.05	1.99E-02	−0.95	2.11E-02
MGI:96995	*Mll1*	Lysine (K)-specific methyltransferase 2A	Calcium ion binding	−1.05	4.97E-03	−0.81	3.37E-02
			Zinc ion binding	
			Chromatin binding	
			Histone methyltransferase activity	
			Regulation of transcription	
MGI:2446229	*Tet3*	Tet methylcytosine dioxygenase 3	Methylcytosine dioxygenase activity	−1.05	1.98E-02	−1.05	6.56E-03
			Oxidoreductase activity	
			Metal ion binding	
			Plays role in the DNA methylation process	
MGI:99948	*Zfhx3*	Zinc finger homeobox 3	GTP binding	−1.06	3.98E-02	−1.12	7.24E-03
			Sequence-specific DNA binding transcription factor activity	
			Zinc ion binding	
MGI:1347464	*Foxg1*	Forkhead box G1	Sequence-specific DNA binding	−1.06	4.30E-02	−1.11	9.22E-03
			Negative regulation of neuron differentiation	
			Regulation of transcription	
			Brain development	
			Forebrain marker (Yahata et al., 2011 no. 296)	
MGI:1919847	*Auts2*	Autism susceptibility candidate 2	Unknown	−1.07	2.00E-02	−1.05	7.45E-03
MGI:1915467	*Prrc2a*	Proline-rich coiled-coil 2A	Unknown	−1.07	4.33E-03	−0.85	2.24E-02
MGI:1917685	*Inf2*	Inverted formin, FH2 and WH2 domain containing	Rho GTPase binding	−1.07	7.70E-03	−0.87	2.60E-02
			actin binding	
MGI:2158663	*Inpp5j*	Inositol polyphosphate 5-phosphatase J	SH3 domain binding	−1.12	2.43E-02	−1.78	6.55E-07
			Hydrolase activity	
			Phosphatase activity	
MGI:2682319	*Mll2*	Lysine (K)-specific methyltransferase 2D	Histone methyltransferase	−1.12	1.90E-03	−1.05	1.48E-03
MGI:3026647	*Flrt1*	Fibronectin leucine rich transmembrane protein 1	Receptor signaling protein activity	−1.18	1.53E-02	−1.25	1.89E-03
MGI:1888520	*Brd4*	Bromodomain containing 4	DNA binding	−1.19	4.97E-03	−1.05	7.64E-03
MGI:1916205	*Srrm4*	Serine/arginine repetitive matrix 4	mRNA binding	−1.22	2.43E-02	−1.25	6.20E-03
			Promotes alternative splicing and inclusion of neural-specific exons in target mRNAs	
MGI:1926106	*Fam163b*	Family with sequence similarity 163, member B	Unknown	−1.27	9.85E-03	−1.60	2.91E-05
MGI:2685951	*Myo16*	Myosin XVI	Motor activity	−1.27	1.57E-03	−1.09	4.62E-03
			ATP-binding	
			Protein phosphatase binding	
MGI:1923304	*Prrc2b*	Proline-rich coiled-coil 2B	Unknown	−1.30	2.37E-05	−1.11	2.28E-04
MGI:1923206	*Srrm2*	Serine/arginine repetitive matrix 2	C_2_H_2_ zinc finger domain binding	−1.33	8.92E-06	−1.04	7.25E-04
			RNA binding involved in pre-mRNA splicing	
MGI:1337080	*Ncor2*	Nuclear receptor co-repressor 2	Chromatin binding	−1.38	5.62E-05	−1.39	1.21E-05
			Regulation of transcription	
			Transcription co-repressor activity	
			Notch binding	
MGI:1306776	*Mtap1a*	Microtubule-associated protein 1 A	Structural molecule activity	−1.44	3.82E-07	−1.62	9.68E-10
			Microtubule assembly	
			Perception of sound	
MGI:104725	*Atn1*	Atrophin 1	Toxin receptor binding	−1.44	2.15E-04	−1.32	3.38E-04
			Transcription co-repressor activity	
MGI:104296	*Nova2*	Neuro-oncological ventral antigen 2	RNA binding	−1.49	4.27E-05	−1.64	7.71E-07
MGI:3613677	*Shank1*	SH3 and multiple ankyrin repeat domains 1	SH3 domain binding	−1.57	9.26E-08	−1.53	7.43E-08
			Identical protein binding	
			Synapse maturation	
MGI:2679002	*Prr12*	Proline-rich 12	DNA binding	−1.59	3.68E-04	−1.68	2.80E-05
MGI:2143886	*Dot1l*	DOT1-like histone H3 methyltransferase	Transcription factor binding	−1.63	3.29E-05	−1.06	1.72E-02
			DNA binding	
			Histone-lysine *N*-methyltransferase activity	
MGI:88107	*Atp1a3*	Sodium/potassium-transporting ATPase subunit alpha-3	Sodium:potassium-exchanging ATPase activity	−1.73	1.31E-10	−1.90	1.70E-13
			ATP-binding	
			Metal ion binding	
			Hydrolase activity	
MGI:98974	*Xist*	Inactive × specific transcripts	Non-protein coding	−1.73	1.31E-10	−0.90	5.70E-03

The biggest differences between aged Tg4–42 and 5XFAD could be detected in the expression of *Gfap* and *Xist*. The intermediate filament protein GFAP encoding gene was found to be four times higher over-expressed in 5XFAD compared to Tg4–42. The non-protein coding RNA *Xist* was twofold less abundant in Tg4–42 as compared to 5XFAD mice. Apart from *Gfap*, *Calmodulin 3*, *Fbxo2*, and *Gpm6a* were also up-regulated in both aged mouse lines.

The functional annotation of the jointly down-regulated genes includes the following gene ontology (GO) (Ashburner et al., 2000) categories: regulation of cell differentiation and anatomical structure development, regulation of gene expression and transcription, histone modification, ion binding and protein methyltransferase activity, nervous system development, and neurogenesis.

Two genes were similarly down-regulated in aged Tg4–42 and 5XFAD but also young 5XFAD animals (Figure 6). First, *Ubqln2* which encodes a member of the ubiquilin family (Ubiquilin 2) that is involved in the protein degrading pathway as it regulates the degradation of ubiquitinated proteins (Ko et al., 2004). Second, the RNA binding protein neuro-oncological ventral antigen 2 encoding gene (*Nova2*).

### Gene expression in aged 5XFAD mice

In aged 5XFAD mice, 131 genes with significant expression changes were identified. While 62 genes were up-regulated, 69 genes were down-regulated (Figure 5B). Eighty-seven of the genes were only found to be altered in aged 5XFAD mice (Table 5), while 36 showed an overlap with aged Tg4–42 mice (Table 4; Figure 6) and eight were also differentially expressed in young 5XFAD mice.

**Table 5:** **List of differentially expressed transcripts in aged 5XFAD mice**.

ID	Gene name	Gene description	GO biological process annotation/functions	log2 Fold change	Adjusted *p*-value
MGI:88228	*C4b*	Complement component 4B	Endopeptidase inhibitor activity	3.31	2.44E-27
			Inflammatory response		
			Complement activation		
			Immune response		
MGI:88225	*C1qc*	Complement component 1, q subcomponent, C chain	Complement activation	3.05	1.84E-23
			Immune response		
MGI:88223	*C1qa*	Complement component 1, q subcomponent, alpha polypeptide	Phosphate transport	2.99	1.31E-24
			Complement activation		
			Immune response		
MGI:107341	*Ctss*	Cathepsin S	Cysteine-type peptidase activity	2.75	5.00E-21
			Hydrolase activity		
			Proteolysis		
			Immune response		
MGI:88224	*C1qb*	Complement component 1, q subcomponent, beta polypeptide	Phosphate transport	2.72	2.22E-19
			Complement activation		
			Immune response		
MGI:1891190	*Ctsz*	Cathepsin Z	Cysteine-type peptidase activity	2.49	1.01E-15
			Hydrolase activity		
MGI:98932	*Vim*	Vimentin	Structural constituent of cytoskeleton	2.27	8.36E-13
			Identical protein binding		
			Apoptotic process		
MGI:96074	*Hexb*	Hexosaminidase B	Cation binding	2.16	9.49E-15
			Protein homodimerization activity		
			Beta-*N*-acetylhexosaminidase activity		
			Protein heterodimerization activity		
MGI:88562	*Ctsd*	Cathepsin D	Aspartic-type endopeptidase activity	2.13	1.66E-16
			Hydrolase activity		
MGI:2148181	*Snora68*	Small nucleolar RNA, H/ACA box 68	Non-coding RNA	2.11	4.37E-14
			Uridine modifications		
MGI:87994	*Aldoa*	Aldolase A, fructose-bisphosphate	Actin binding	2.03	1.32E-15
			Fructose-bisphosphate aldolase activity		
			Cytoskeletal protein binding		
			Tubulin binding		
			Glycolysis		
MGI:88127	*B2m*	Beta-2 microglobulin	MHC class I receptor activity	1.86	5.03E-10
			Cellular defense response		
			Innate immune response		
MGI:108046	*Laptm5*	Lysosomal-associated protein transmembrane 5	Transmembrane transport	1.86	1.32E-07
MGI:1333815	*Cx3cr1*	Chemokine (C-X3-C motif) receptor 1	Chemokine receptor activity	1.66	9.43E-07
			G-protein coupled receptor activity		
			Transmembrane protein		
			Signal transduction		
MGI:99554	*Lgals3bp*	Lectin, galactoside-binding, soluble, 3 binding protein	Scavenger receptor activity	1.66	1.19E-05
			Isomerase activity		
			Cellular defense response		
			Signal transduction		
MGI:1921298	*4632428N05Rik*	RIKEN cDNA 4632428N05 gene	Receptor activity	1.63	1.48E-06
MGI:96614	*Itgb5*	Integrin beta 5	Integrin binding	1.56	1.05E-06
			Receptor activity		
			Cell adhesion		
MGI:1914877	*Olfml3*	Olfactomedin-like 3	Scaffold protein	1.53	1.36E-04
MGI:96073	*Hexa*	Hexosaminidase A	Beta-*N*-acetylhexosaminidase activity	1.51	3.85E-06
			Protein heterodimerization activity		
			Hydrolase activity		
MGI:1339758	*Csf1r*	Colony -stimulating factor 1 receptor	Macrophage colony-stimulating factor Receptor activity	1.45	1.58E-06
			Protein homodimerization activity		
			ATP-binding		
			Immune response		
MGI:107387	*Aqp4*	Aquaporin 4	Porin activity	1.42	8.90E-07
			Water transmembrane transporter activity		
MGI:1918089	*P2ry12*	Purinergic receptor P2Y, G-protein coupled 12	ADP receptor activity	1.40	2.68E-04
			Guanyl-nucleotide exchange factor activity		
			G-protein coupled adenosine receptor activity		
			Signal transducer activity		
MGI:1278340	*Rpl21*	Ribosomal protein L21	Structural constituent of ribosome	1.37	2.20E-04
			RNA binding		
MGI:107286	*Man2b1*	Mannosidase 2, alpha B1	Carbohydrate binding	1.35	4.77E-05
			Alpha-mannosidase activity		
			Hydrolase activity		
MGI:1096881	*Eef1a1*	Eukaryotic translation elongation factor 1 alpha 1	GTPase activity	1.32	6.22E-06
			Translation elongation factor activity		
			Regulation of transcription		
MGI:1915213	*Npc2*	Niemann Pick type C2	Cholesterol binding	1.27	1.20E-03
			Enzyme binding		
MGI:88561	*Ctsb*	Cathepsin B	Cysteine-type peptidase activity	1.25	1.21E-05
			Hydrolase activity		
			Immune response		
MGI:88564	*Ctsl*	Cathepsin L	Cysteine-type peptidase activity	1.23	3.42E-04
			Hydrolase activity		
			Histone binding		
			Immune response		
MGI:1934664	*Rpph1*	Ribonuclease P RNA component H1	Endoribonuclease activity	1.23	1.21E-05
MGI:1920174	*Anln*	Anillin	Actin binding	1.22	1.53E-03
			Phospholipid binding		
MGI:95832	*Grn*	Granulin	Growth factor activity	1.21	1.00E-03
			Cytokine activity		
			Signal transduction		
MGI:98729	*Tgfbr2*	Transforming growth factor, beta receptor II	ATP-binding	1.18	4.94E-03
			Transmembrane receptor protein serine/threonine kinase activity		
			Transferase activity		
			Receptor activity		
			SMAD binding		
			Signal transduction		
MGI:894320	*Prdx6*	Peroxiredoxin 6	Glutathione peroxidase activity	1.17	2.21E-04
			Oxidoreductase activity		
			Antioxidant activity		
			Response to oxidative stress		
MGI:1921305	*Plce1*	Phospholipase C, epsilon 1	Guanyl-nucleotide exchange factor activity	1.15	5.42E-03
			Calcium ion binding		
			Receptor signaling protein activity		
			Hydrolase activity		
			Signal transducer activity		
MGI:107357	*Inpp5d*	Inositol polyphosphate-5-phosphatase D	SH3 domain binding	1.14	1.96E-02
			PTB domain binding		
			Hydrolase activity		
			Signal transducer activity		
			Immune response		
MGI:1330838	*Lgmn*	Legumain	Cysteine-type endopeptidase activity	1.07	1.82E-03
			Peptidase activity		
			Immune response		
			Hydrolase activity		
MGI:1917329	*Golm1*	Golgi membrane protein 1	Protein modification	1.03	2.85E-02
			Nucleus organization	
MGI:88385	*Cfh*	Complement component factor h	Heparin binding	1.02	4.65E-02
			Heparan sulfate proteoglycan binding		
			Complement activation		
			Immune response		
MGI:95640	*Gapdh*	Glyceraldehyde-3-phosphate dehydrogenase	Microtubule binding	1.01	3.07E-03
			NADP binding		
MGI:1924096	*Rps9*	Ribosomal protein S9	Structural constituent of ribosome	1.01	1.59E-02
			RNA binding		
			Translation regulator activity		
MGI:97171	*Mt1*	Metallothionein 1	Organic cyclic compound binding	1.01	3.08E-03
			Hormone binding	
			Copper ion binding	
MGI:97591	*Pkm*	Pyruvate kinase, muscle	Magnesium ion binding	1.00	1.56E-03
			Pyruvate kinase activity	
			ATP-binding	
MGI:88423	*Clu*	Clusterin	ATPase activity	0.96	2.50E-03
			Ubiquitin -protein ligase binding	
			Misfolded protein binding	
			Immune response	
MGI:1338892	*Padi2*	Peptidyl arginine deiminase, type II	Protein-arginine deiminase activity	0.95	4.86E-02
			Calcium ion binding	
			Hydrolase activity	
			Immune response	
MGI:1915472	*Tubb4b*	Tubulin, beta 4B class IVB	Structural molecule activity	0.93	2.03E-02
			GTPase activity	
			Double-stranded RNA binding	
			Structural constituent of cytoskeleton	
			Unfolded protein binding	
MGI:2445114	*Pisd*	Phosphatidylserine decarboxylase	Lyase activity	0.93	1.94E-02
MGI:96247	*Hsp90ab1*	Heat shock protein 90 alpha (cytosolic), class B member 1	Unfolded protein binding	0.90	6.13E-03
			GTP binding	
			ATP-binding	
			Double-stranded RNA binding	
			Ion channel binding	
			Immune response	
			Negative regulation of neuron apoptotic process	
MGI:1925017	*Ermn*	Ermin, ERM-like protein	Actin filament binding	0.90	3.02E-02
MGI:105959	*Cox8a*	Cytochrome-*c* oxidase subunit VIIIa	Cytochrome-*c* oxidase activity	0.88	1.52E-02
MGI:96748	*Lamp2*	Lysosomal-associated membrane protein 2	Membrane glycoprotein	0.88	2.94E-02
			TRNA ligase activity	
			ATP-binding	
			Hemostasis	
MGI:97748	*Ctsa*	Cathepsin A	Enzyme activator activity	0.86	3.15E-02
			Serine-type carboxypeptidase activity	
			Hydrolase activity	
MGI:103099	*Cox6a1*	Cytochrome-*c* oxidase subunit VIa polypeptide 1	Cytochrome-*c* oxidase activity	0.85	2.02E-02
MGI:1346074	*Fxr2*	Fragile × X mental retardation, autosomal homolog 2	RNA binding	0.84	3.11E-02
			Identical protein binding	
MGI:99607	*Abca1*	ATP-binding cassette, sub-family A (ABC1), member 1	Apolipoprotein binding	0.82	4.37E-02
			Phospholipid binding	
			Cholesterol binding	
			ATP-binding	
MGI:88252	*Calr*	Calreticulin	Iron ion binding	0.81	3.02E-02
			Calcium ion binding	
			Hormone binding	
			mRNA binding	
			Regulation of transcription	
			Signal transduction	
			Immune system	
MGI:98467	*Syp*	Synaptophysin	Transporter activity	0.79	2.74E-02
			Calcium ion binding	
			Cholesterol binding	
			Syntaxin-1 binding	
			SH2 domain binding	
			Synaptic vesicle maturation	
			Synaptic transmission	
MGI:98373	*Sparc*	Secreted acidic cysteine rich glycoprotein	Extracellular matrix binding	0.78	4.37E-02
			Calcium ion binding	
			Signal transduction	
			Hemostasis	
MGI:1096398	*Cd81*	CD81 antigen	MHC class II protein complex binding	0.77	4.93E-02
			Regulation of immune response	
MGI:1915347	*Dynll2*	Dynein light chain LC8-type 2	Cytoskeletal protein binding	−0.86	2.10E-02
			Motor activity	
MGI:1277955	*Bsn*	Bassoon	Metal ion binding	−0.87	1.10E-02
			Synaptic transmission	
MGI:3648294	*Tnrc18*	Trinucleotide repeat containing 18	DNA binding	−0.87	3.34E-02
MGI:2145310	*Rnf44*	Ring finger protein 44	Zinc ion binding	−0.89	3.49E-02
MGI:103291	*Rai1*	Retinoic acid induced 1	Zinc ion binding	−0.90	2.82E-02
			DNA binding	
			Transcription factor	
MGI:1096362	*Nrxn2*	Neurexin II	Cell adhesion molecule binding	−0.90	1.19E-02
			Calcium channel regulator activity	
			Metal ion binding	
			Synaptic transmission	
MGI:1337000	*Rn45s*	45S pre-ribosomal 5	Non-coding RNA	−0.90	5.41E-03
MGI:88106	*Atp1a2*	ATPase, Na+/K+ transporting, alpha 2 polypeptide	Sodium:potassium-exchanging ATPase activity	−0.90	5.39E-03
			Metal ion binding	
			Hydrolase activity	
			ATP-binding	
MGI:96667	*Kcnc1*	Potassium voltage -gated channel, shaw-related sub-family, member 1	Rectifier potassium channel activity	−0.92	1.07E-02
			Voltage-gated ion channel activity	
			Synaptic transmission	
MGI:1347488	*Foxk1*	Forkhead box K1	Transcription regulation	−0.94	3.02E-02
			Cell differentiation	
			DNA binding	
			Mg-ion binding	
MGI:106190	*Bcl11a*	B cell CLL/lymphoma 11A (zinc finger protein)	B cell differentiation	−0.96	3.73E-02
			T cell differentiation	
			Regulation of transcription	
MGI:1321395	*Ltbp4*	Latent transforming growth factor beta binding protein 4	Growth factor binding	−0.96	4.59E-02
			Hormone secretion	
			Regulation of cell differentiation	
MGI:2176606	*Scrt1*	Scratch homolog 1, zinc finger protein	Transcription regulation	−0.96	2.09E-02
MGI:1925589	*Ttyh3*	Tweety homolog 3	Chloride channel activity	−0.96	7.91E-03
			Transmembrane transport	
MGI:2444218	*Ahdc1*	AT hook, DNA binding motif, containing 1	DNA binding	−0.97	1.84E-02
MGI:98460	*Syn1*	Synapsin I	Neurotransmitter secretion	−0.98	3.98E-03
			Synaptic vesicle transport	
MGI:95617	*Gabra5*	Gamma-aminobutyric acid (GABA) A receptor, subunit alpha 5	GABA-receptor	−0.99	1.52E-02
			Chloride transport	
			Gamma-aminobutyric acid signaling pathway	
MGI:2143099	*AI593442*	Expressed sequence AI593442	Unclassified	−1.00	4.30E-03
MGI:1346031	*Tshz1*	Teashirt zinc finger family member 1	Transcription factor	−1.00	1.73E-02
			DNA binding	
MGI:2444817	*C530008 M17Rik*	RIKEN cDNA C530008M17 gene	Unknown	−1.00	2.26E-02
MGI:2441680	*Tmem8b*	Transmembrane protein 8B	Cell cycle regulation	−1.00	2.27E-02
			Cell matrix adhesion	
MGI:109169	*Epas1*	Endothelial PAS domain protein 1	Regulation of transcription	−1.01	6.32E-03
			Angiogenesis	
			Transcription	
			Signal transduction	
			Cellular stress response	
MGI:1351323	*Snord33*	Small nucleolar RNA, C/D box 33	Unknown	−1.04	4.35E-02
MGI:107363	*Stxbp1*	Syntaxin binding protein 1	Release of neurotransmitters via syntaxin regulation	−1.04	7.75E-04
			Vesicle transport	
			Exocytosis	
			Regulation of insulin secretion	
MGI:2443847	*Sdk2*	Sidekick homolog 2	Chemotaxis	−1.04	2.43E-02
			Protein targeting	
			Cell adhesion	
MGI:1919559	*Tmem158*	Transmembrane protein 158	Ras pathway	−1.06	2.27E-02
MGI:102858	*Fosl2*	Fos-like antigen 2	Regulation of transcription	−1.10	1.07E-02
			Cell regulation	
			Fibroblasten proliferation	
MGI:2686934	*Zfhx2*	Zinc finger homeobox 2	DNA binding	−1.11	6.32E-03
			Transcriptional factor activity	
MGI:96434	*Igf2*	Insulin-like growth factor 2	Hormone activity	−1.11	4.47E-03
			Growth factor activity	
			Cell proliferation	
			Regulation of cell cycle	
			Protein metabolism	
			Hemostasis	
			Signal transduction	
MGI:2444034	*9530091C08Rik*	RIKEN cDNA 9530091C08 gene	Unclassified non-coding RNA gene	−1.14	1.10E-02
MGI:1313277	*Vamp2*	Vesicle-associated membrane protein 2	Vesicle mediate transport	−1.14	2.79E-04
			Synaptic vesicle exocytosis	
			Regulation of insulin secretion	
MGI:1890616	*Scube1*	Signal peptide, CUB domain, EGF-like 1	Inflammatory response	−1.19	1.28E-03
			Endothelial cell differentiation	
MGI:2444210	*Nr1d1*	Nuclear receptor sub-family 1, group D, member 1	Transcription factor	−1.23	6.34E-04
			Insulin secretion	
			Metabolic processes	
			Inflammatory processes	
MGI:2444521	*Rnf165*	Ring finger protein 165	Zinc ion binding	−1.25	1.05E-02
MGI:1351339	*Grm2*	Glutamate receptor, metabotropic 2	Synaptic transmission	−1.27	2.12E-03
			Glutamate secretion	
MGI:102703	*Gng4*	Guanine nucleotide binding protein (G-protein), gamma 4	Signal transduction	−1.42	7.64E-05
			GTPase activity	
			Hemostasis	
			Synaptic transmission	
			Glucagon response	
			Transmembrane transport of small molecules	
MGI:95295	*Egr1*	Early growth response 1	Transcriptional regulator	−1.55	6.15E-07
			Immune response		
			T cell differentiation		

A notable group DEGs is involved in immune system processes and inflammation (according to the GO annotation). These are, among others, innate immune response and adaptive immune response, immune effector processes, activation and regulation of immune response as well as immune system development.

Furthermore, DEGs were also involved in cell communication and system development, signal transduction, synaptic transmission as well as regulation of gene expression and transcription.

### Genes similarly expressed in both young and aged 5XFAD mice

Eight genes were found to be differentially expressed in both young and aged 5XFAD mice (Figure 6). Of these genes, four were up-regulated and four down-regulated. The up-regulated genes are the ribosomal protein *Rpl21*, *Aldolase A*, *Snora68*, and the ribonuclease P RNA component H1. *Ubqln2*, *Nova2*, *Atp1a2*, and *Rn45s* showed reduced expression.

### Validation of differentially expressed genes identified by RNA-Seq using real-time PCR

The quality of the isolated RNA is crucial for obtaining reliable qRT-PCR results. Therefore, the quality of the RNA samples isolated from the mice brains was evaluated by assessing the integrity and purity of the RNA. All samples displayed A260/A230 ratios greater than 1.8 and A260/A280 ratios higher than 2.0 (data not shown) indicating an acceptable RNA purity.

**Figure 7:**
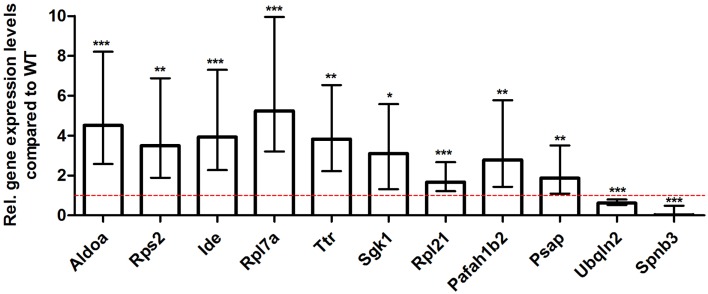
**Validation of young 5XFAD deep sequencing results through quantitative real-time polymerase chain reaction (qRT-PCR) analysis**. To confirm the deep sequencing data, qRT-PCR experiments for various genes were performed on young 5XFAD and age-matched WT mice. Expression levels of 5XFAD mice were compared to age-matched WT animals (dashed red line represents WT standard). Normalization was performed against the housekeeping gene β-Actin. ***p < 0.001; **p < 0.01; *p < 0.05; *m* age in months; *n* = 4–5 per group.

**Figure 8:**
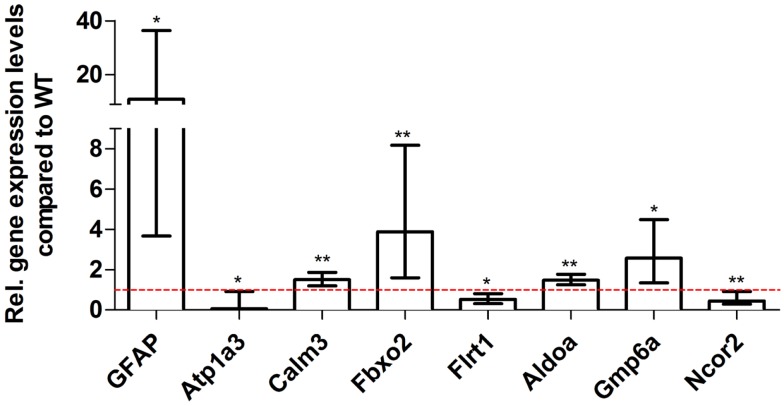
**Validation of aged 5XFAD deep sequencing results through quantitative real-time polymerase chain reaction (qRT-PCR) analysis**. To confirm the deep sequencing data, qRT-PCR experiments for various genes were performed on aged 5XFAD and age-matched WT mice. Expression levels of 5XFAD mice were compared to age-matched WT animals (dashed red line represents WT standard). Normalization was performed against the housekeeping gene β-Actin. **p < 0.01; *p < 0.05; *m* age in months; *n* = 4–5 per group.

**Figure 9:**
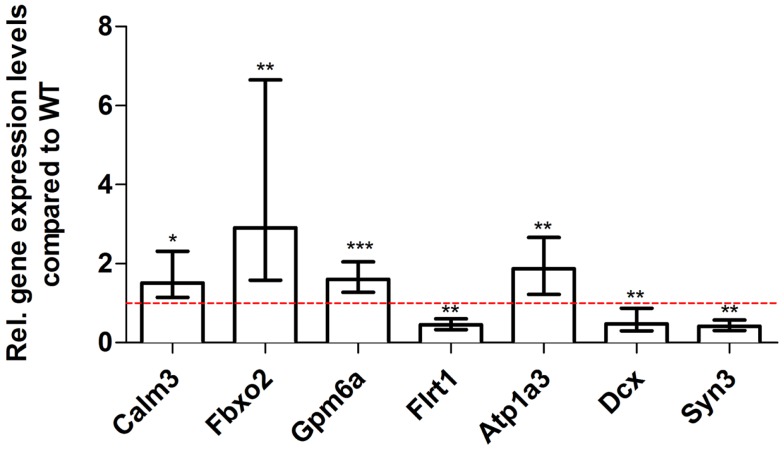
**Validation of aged Tg4–42 deep sequencing results through quantitative real-time polymerase chain reaction (qRT-PCR) analysis**. To confirm the deep sequencing data, qRT-PCR experiments for various genes were performed on aged Tg4–42 and age-matched WT mice. Expression levels of Tg4–42 mice were compared to age-matched WT animals (dashed red line represents WT standard). Normalization was performed against the housekeeping gene β-Actin. ***p < 0.001; **p < 0.01; *p < 0.05; *m* age in months; *n* = 4–5 per group.

For young 5XFAD (Figure 7), aged 5XFAD (Figure 8), and Tg4–42 (Figure 9) mice at least seven DEGs were randomly selected and validated using qRT-PCR. For all genes, the qRT-PCR analysis revealed expression patterns similar to the deep sequencing results.

## Discussion

The transcriptome includes all RNA transcripts expressed in a given tissue and renders a profile of genes that are expressed at the studied time point. Altered gene expression profiles may therefore provide information about the genes and mechanisms involved in the molecular pathogenesis of diseases like AD and ultimately promote the search for new therapeutic drugs.

### Advantages of mRNA deep sequencing

Microarrays were used in the past as a standard technique for transcriptome profiling. The method has been proven to be valuable to quantify simultaneously large numbers of mRNA transcripts (Courtney et al., 2010). Commercially available microarrays can be used to analyze up to 15,000–30,000 different mRNAs and facilitate genome-wide gene expression profiling (Altar et al., 2009). Oligonucleotide and cDNA microarrays are both affordable and offer a high-throughput approach.

However, due to the use of indirect signal detection by hybridization, microarray techniques possess several limitations (Courtney et al., 2010). These include reliance upon knowledge of already known sequences, poor range of quantification, and relatively low sensitivity and specificity (Choi et al., 2013). Furthermore, the non-specific binding of samples make the detection of low expressed transcripts against the background noise difficult (Sutherland et al., 2011) and unsuitable for the quantification of over- and under-expressed genes with fold changes smaller than two (Wang et al., 2009). van Bakel et al. (2010) reported that hybridization signals from microarrays can lead to a high number of false positive signals especially from transcripts with low expression levels.

Several microarray studies on amyloid mouse models for AD have been reported (Stein and Johnson, 2002; Dickey et al., 2003; Wu et al., 2006; Selwood et al., 2009; Wirz et al., 2013). The transgenic models included APP/PS1_ΔEx9_, PDAPP, Tg2576, and combinations with different mutant PSEN-1 genetic variants. All of these transgenic lines represent models for familial AD and abundant plaque formation without severe neuron loss. Therefore, we compared two models that do show a robust behavioral deficit and in addition harbor a significant neuron loss.

RNA-Seq allows to cope with many of the problems described for microarrays and has a number of advantages over microarray technology. Most importantly, deep sequencing does not rely on known genome sequence data and therefore novel transcripts can be detected (Courtney et al., 2010). It is possible to detect billions of nucleotide information within a single experiment (Cheng et al., 2013). Furthermore, problems with saturation and background signal do not exist as each molecule is individually sequenced and mapped to unique regions of the genome. RNA-Seq offers a larger dynamic range than microarray technology as no upper or lower levels exist in this quantification technique (Courtney et al., 2010). In comparison to microarrays, deep sequencing has a low false positive rate and is moreover highly reproducible (Nagalakshmi et al., 2008).

### 5XFAD, a model for familial Alzheimer’s disease

Using deep sequencing technology, we analyzed the RNA profiles from the two AD models 5XFAD and Tg4–42 (Table 6). We compared these two models, because they show a robust behavioral deficit and in addition develop a significant neuron loss.

**Table 6:** **Comparison of the two transgenic mouse models 5XFAD and Tg4–42**.

Features	5XFAD	Tg4–42
Mutations	APP695 (Swedish, Florida, London)	None
	PSEN-1 (M146L and L286V)	
Genetic background	C57Bl6	C57Bl6
Transient intraneuronal Aβ	Yes	Yes
Prevalence of Aβ variants	Aβ1–42 > 1–40 > 4–42 > pyroglutamate3-42	only Aβ4–42
Plaques	Plaque deposits starting at 3 months	None
Neuron loss	38% loss in cortical layer 5	49% loss in CA1
Gliosis	Yes	Yes
Behavioral deficits	Yes	Yes

5XFAD is a model for familial AD that shows massive and early plaque formation, intraneuronal Aβ aggregation, behavioral deficits, and neuron loss in the neocortical layer 5 and subiculum (Oakley et al., 2006; Jawhar et al., 2010). In the 5XFAD model, many molecular pathways are altered due to mutant APP and PS1 over-expression leading to massive elevation of Aβ_1–42_, Aβ_1–40_, Aβ_4–42_, pyroglutamate Aβ_pE3–42_, and Aβ_3–42_ (Wittnam et al., 2012). The consequence of this is that 5XFAD harbor soluble forms of full-length and diverse N-truncated Aβ species that are also found precipitated in plaques.

### Tg4–42, a model for sporadic Alzheimer’s disease

*In vitro* and *in vivo* analysis of amyloid deposits in AD revealed N- and C-terminal variants of the Aβ peptide (Masters et al., 1985; Prelli et al., 1988; Miller et al., 1993). Masters et al. (1985) discovered that the majority (64%) of the peptides in amyloid plaques of AD begin with a phenylalanine residue corresponding to position 4 of the full-length sequence. Moreover, they detected dimeric and tetrameric Aβ aggregates from the HPLC separations of plaques from AD having the same ragged NH_2_-terminal ends. The importance of Aβ_4–42_ was later supported by the finding that it represents a dominant fraction in the hippocampus and cortex of AD patients using immunoprecipitation and mass spectrometry (Portelius et al., 2010).

In order to investigate the long-lasting neurotoxic effect of Aβ_4–42_, we recently generated the novel mouse model Tg4–42 expressing exclusively Aβ_4–42_ (Bouter et al., 2013). Tg4–42 mice develop severe hippocampal neuron loss and memory deficits that correlate well with the hippocampus-specific intraneuronal expression of Aβ_4–42_. These findings are corroborated by previous mouse models expressing full-length mutant APP. For example, APP/PS1KI mice exhibit neuron loss in the CA1 region of the hippocampus (Casas et al., 2004; Breyhan et al., 2009), the frontal cortex (Christensen et al., 2008), and in distinct cholinergic nuclei (Christensen et al., 2010). The APP/PS1KI model is characterized by age-dependent accumulation of heterogeneous N-terminal truncated Aβ peptides with Aβ_4–42_ being one of the most abundant variants (Casas et al., 2004). In 5XFAD mice, a heterogeneous mixture of full-length, N-truncated and modified Aβ peptides, including Aβ_4–42_, was also found (Wittnam et al., 2012). Hence, the pathological events observed in the APP/PS1 KI and 5XFAD mouse models might be at least partly triggered by N-terminal truncated Aβ_4–42_.

### Learning and memory deficits in 5XFAD and Tg4–42 mice

In the present work, we could show that Tg4–42 mice and 5XFAD mice feature comparable learning and memory deficits. Both mouse lines exhibited age-dependent spatial reference memory deficits as assessed by the Morris water maze. Aged Tg4–42 and 5XFAD mice have also been tested in the CFC paradigm and exhibited deficits in this hippocampus-dependent memory tasks. Tg4–42 and 5XFAD mice displayed hippocampus-dependent memory deficits similar to those of other AD transgenic models (Chen et al., 2000; Stover and Brown, 2012; Kishimoto et al., 2013).

Classical fear conditioning is assumed to be highly dependent on the hippocampus (Bast et al., 2003). Phillips and LeDoux (1992) reported that lesions of the hippocampus interfered with CFC but not with cue and TFC. In contrast, a functional amygdala is required for appropriate fear conditioning for both context and tone. Moreover, anxiety behavior was claimed to correlate with the presence of intraneuronal Aβ in the amygdala (España et al., 2010). These observations are in agreement with the impairment in conditioned learning in response to a tone stimulus of aged 5XFAD mice, but not of age-matched Tg4–42 animals.

### Deep sequencing in 5XFAD and Tg4–42 mice

In order to detect gene expression changes in the two AD mouse models, deep sequencing analysis was performed on young as well as aged 5XFAD and Tg4–42 mice. A wide range of DEGs could be identified in aged Tg4–42 as well as in young and aged 5XFAD mice compared to age-matched wildtype controls, respectively. Even though, the potential for false positive results cannot be eliminated completely, more than 25 transcript changes detected by RNA-Seq could be successfully validated by qRT-PCR and therefore validated the deep sequencing results. Furthermore, the detection of the transgenic human PSEN-1 and APP sequences in young and aged 5XFAD mice through deep sequencing is also an indication for the quality of the method.

The expression changes detected in the transgenic mice give a broad picture of the profound physiological changes that accompany the neuron loss and the detected memory deficits in 5XFAD and Tg4–42 mice. Some of the DEGs have been reported before, while many genes are described for the first time in the context of AD. The observed parallel expression of these genes now offers new perspectives in understanding the pathology of AD.

### Differentially expressed genes in young 5XFAD mice

In young 5XFAD mice, a substantial number of genes is differentially expressed prior to robust amyloid deposition and neuron loss. The 19 DEGs encoded proteins from diverse functional categories, including translation, glycolysis, and ATP-binding, kinases and hydrolases. The 5XFAD model has been reported to develop plaque deposition starting already at the age of 3 months (Jawhar et al., 2010). Intraneuronal Aβ is evident at 1.5 months of age, just before the first appearance of amyloid deposits at 2 months of age (Oakley et al., 2006).

The data of young 5XFAD mice elucidate the expression profile at the commencement of plaque formation and before learning and memory deficits are apparent. Several DEGs that are involved in the clearance of Aβ: transthyretin (*Ttr*) (Li and Buxbaum, 2011) and insulin-degrading enzyme (*Ide*) (Farris et al., 2003; Miners et al., 2009) are found up-regulated.

No DEGs were detected in young Tg4–42 mice, which suggest that the pathology is weak at that age and points to a later onset of the pathological events that underlie the phenotypic changes observed at later ages.

### Common molecular signature of Tg4–42 and 5XFAD mice

Interestingly, 36 genes were differentially expressed in both mouse models indicating common disease pathways associated with behavioral deficits and neuron loss occurring in these mouse models. Nearly half of the DEGs in aged Tg4–42 were also differentially expressed in 5XFAD mice.

Many of the genes that showed differential regulation in 5XFAD alone belong to neuroinflammatory processes typically found associated with plaques. As Tg4–42 mice do not develop any plaques, but massive neuron loss, we assume that the genes isolated in both models and those in Tg4–42 alone are defining the molecular signature underlying memory decline in this mouse model for AD.

The DEGs that were found in both models fall in a broad range of functional categories: regulation of cell differentiation and anatomical structure development, regulation of gene expression and transcription, histone modification, ion binding and protein methyltransferase activity, nervous system development, and neurogenesis.

Together with *Calm3*, *Fbxo2*, and *Gpm6a* only *Gfap* was found to be up-regulated in both aged 5XFAD and Tg4–42 mice. The astrocyte marker glial fibrillary acidic protein gene (*Gfap*) was found to be similarly up-regulated in both mouse lines. Increased astrogliosis was previously described in both mouse lines (Oakley et al., 2006; Bouter et al., 2013). Increased astrogliosis, measured by GFAP concentration, is also found in cortex, thalamus, brainstem, and cerebellum in AD brains (Delacourte, 1990).

Next to the up-regulated genes, 32 genes were commonly down-regulated in aged transgenic mice compared to WT. Among others *Lrp1* was altered. Kanekiyo et al. (2013) demonstrated that receptor-mediated endocytosis in neurons by LRP1 plays a critical role in Aβ clearance in the brain.

Decreased levels of *Shank1* RNA were found in both mouse lines. The levels of the post-synaptic proteins SHANK1 and SHANK3 were also regulated in patients with AD and in the brains of amyloid precursor protein transgenic mice. It has been proposed that Aβ reduces Shank levels in the dendrites (Pham et al., 2010).

The gene coding for the lysine (K)-specific methyltransferase 2D (*Mll2*), also known as *Kmt2b*, that is highly expressed throughout development as well as in adult tissue (Glaser et al., 2006) is down-regulated in aged 5XFAD and Tg4–42. Kerimoglu et al. (2013) showed that mice lacking *Mll2* in the adult forebrain displayed impaired hippocampus-dependent memory function. Furthermore, the loss of MLL2 leads to down-regulation of genes implicated in neuronal plasticity. 5XFAD and Tg4–42 also showed hippocampus-dependent memory impairments. The down-regulation of *Mll2* that is reported to be crucial for memory consolidation and regulation of hippocampal plasticity genes is well in line with our findings.

### Differentially expressed genes in aged Tg4–42 mice

#### Up-regulated genes in aged Tg4–42 mice

Twenty genes were solely differentially expressed in aged Tg4–42. Among these, only the genes *Uqcc2*, *Beta-S*, and *Kif1a* were found to be up-regulated. *Kif1a* is a member of the kinesin family (KIFs) (Takemura et al., 1996) and has previously been connected to AD (Kondo et al., 2012). These microtubule-based motor proteins transport membrane organelles, mRNA, and proteins (Hirokawa et al., 2009). By transporting those complexes, KIFs play important roles in neuronal function and plasticity as well as morphogenesis and survival (Hirokawa et al., 2010). In neurons, KIF1A transports components of synaptic vesicles containing synaptic vesicle proteins such as synaptophysin and synaptotagmin (Hirokawa et al., 2010). Recently, Kondo et al. (2012) could show that an up-regulation of KIF1A contributes to synaptogenesis in the hippocampus.

#### Down-regulated genes in aged Tg4–42 mice

The 17 genes that were significantly down-regulated in aged Tg4–42 are involved in diverse biological processes. These include regulation of gene expression, nervous system development, cell communication, metal ion transport, neurogenesis, and regulation of synaptic plasticity. The gene encoding nerve growth factor inducible protein (VGF), which is down-regulated in aged Tg4–42, is a neurosecretory protein that is solely expressed in neurons (van den Pol et al., 1994). Adult VGF is detected in several areas in the brain including the olfactory system, cerebral cortex, hypothalamus, and hippocampus as well as the adrenal medulla and motor neurons of the spinal cord (van den Pol et al., 1994; Snyder and Salton, 1998; Thakker-Varia and Alder, 2009). Several groups proposed VGF as a potential biomarker for AD (Carrette et al., 2003; Jahn et al., 2011). They detected lower protein levels of VGF in the cerebrospinal fluid (CSF) of AD patients compared to healthy controls.

Another notable down-regulated gene in aged Tg4–42 mice codes for doublecortin (*Dcx*). Doublecortin is a microtubule-associated protein that is expressed in migrating neuronal precursors of the developing CNS and immature neurons (Couillard-Despres et al., 2005). Human DCX is often used as a marker for neurogenesis (Couillard-Despres et al., 2005; Verwer et al., 2007). In AD mouse models expressing mutant forms of APP or PSEN-1, neurogenesis was found to be impaired. Aβ was found to disrupt neurogenesis in the subventricular zone and the hippocampus in these mice (Haughey et al., 2002a,b). Jin et al. (2004) however described increased levels of doublecortin in the hippocampus of AD patients brains and therefore suggested that neurogenesis is increased in AD hippocampus.

The pathology of AD has recently been linked to the deregulation of cyclin-dependent kinase 5 (CDK5) (Shukla et al., 2012). CDK5 is regulated by the neuron-specific cyclin-related proteins p35 (CDK5R1) and p39 (CDK5R2). Activated CDK5 plays an important role in neurogenesis, synaptic plasticity and neuronal survival (Nikolic et al., 1996; Tan et al., 2003; Shukla et al., 2012). CDK5 phosphorylates tau and the CDK5 complex is involved in posttranslational modification of APP and PSEN (Rademakers et al., 2005). Various neurotoxic events, including oxidative stress and elevated Aβ levels, result in calpain cleavage of the regulatory proteins p39 and p35. The resulting C-terminal truncated proteins p29 and p25 lead to hyperactivation and mislocalization of CDK5. The introduction of p25 in primary neurons leads to the deregulation of CDK5 causing among others phosphorylation of tau and neuronal cell death (Cruz and Tsai, 2004; Rademakers et al., 2005). It can be hypothesized that the over-expression of Aβ_4–42_ in Tg4–42 mice stimulates activation of calpain and therefore down-regulation of Cyclin-dependent kinase 5 activator 2.

It is notable that several DEGs in aged Tg4–42 mice have an ion binding function. The proteins ZMIZ2 and ZFP609 bind to zinc ions while Beta-S is an iron ion binding protein. Furthermore, the metal ion binding proteins MLL1, ZFHX3, SRRM2, and ATP1A are down-regulated in both aged Tg4–42 and 5XFAD mice. The binding targets zinc and iron, in addition to copper, have been shown to be involved in the pathology of AD. Zinc promotes the aggregation of Aβ (Watt et al., 2010) and was found to be enriched in AD plaques (Lovell et al., 1998; Leskovjan et al., 2011; Roberts et al., 2012). While the overall Zn level in the aging brain is relatively constant, the zinc transporter ZnT3 has been shown to decrease with age (Roberts et al., 2012). Furthermore, disruption of zinc homeostasis in the brain leads to synaptic and memory deficits (Watt et al., 2010). Aged 5XFAD mice also displayed a variety of DEGs involved in metal binding, for example *Bsn*, *Rnf44*, *Rai1*, *Atp1a2*, and *Rnf165*.

### Differentially expressed genes in aged 5XFAD mice

#### Inflammatory processes

In aged 5XFAD mice 131 genes with significant expression changes were identified. Eighty-seven of these genes were only found to be altered in this mouse line and not in Tg4–42. Compared to aged Tg4–42 mice, a significant larger number of genes were differentially expressed in aged 5XFAD mice.

Recently, Upadhaya et al. (2013) suggested somatic versus neuritic mechanism by which Aβ may cause neurodegeneration in APP48 and APP23 transgenic mice, respectively. The authors defined the somatic type of neurodegeneration as intraneuronal accumulations of Aβ that are produced independent of APP. In contrast to the APP48 model, the Tg4–42 mice did not develop such a dendritic pathology (Bouter et al., 2013). This may be due to the different signal peptides used in APP48 (preproenkephalin) and Tg4–42 (thyreotropin-releasing hormone). The signal peptide of Tg4–42 ensures the routing through the secretory pathway allowing the release of the peptide from neurons.

The neuritic type of neurodegeneration linking APP-derived extra- and intracellular Aβ aggregation may be similar between APP23 and 5XFAD mice. Hence, the DEGs observed in 5XFAD and Tg4–42 mice could be partly explained by the different mechanism by which Aβ causes neurodegeneration in these two models.

A large number of DEGs is involved in regulation of immune system processes and inflammation. The respective transcripts are involved among others in adaptive immune response, regulation, and activation of immune response as well as immune system development. Inflammatory processes in the brain are a well-described feature of AD. It has been shown that plaque deposition in AD brains is associated with chronic inflammation characterized by increased inflammatory cytokine expression and activation of microglia, astrocytes, and complement factors (Akiyama et al., 2000). Inflammation is thought to be a downstream process appearing after Aβ plaques, NFT, and neuron degeneration (Arnaud et al., 2006). 5XFAD mice display distinct neuroinflammatory features. The number of reactive astrocytes and microglia increases proportionally to the amyloid burden in this mouse line (Oakley et al., 2006; Kalinin et al., 2009).

5XFAD mice also show a dramatic increase in Aβ42 in comparison to Aβ40. This results in an early pathology onset with plaque deposition seen as early as 3 months of age. The plaque pathology increases dramatically in an age-dependent manner (Oakley et al., 2006; Jawhar et al., 2010). Aggregation of Aβ results in activated microglia and induces the production of reactive-oxygen species, pro-inflammatory cytokines, chemokines, and prostaglandines leading to degenerative changes in neurons (Akiyama et al., 2000).

A large number of DEGs in aged 5XFAD mice have a role in inflammatory pathways (including *Scube1* and *Nr1d1*). Strikingly, four genes of the complement system (*C4b*, *C1qa*, *C1qb*, and *C1qc*) are highly up-regulated in 12-month-old 5XFAD mice. Complement activation is a major inflammatory process and is thought to be activated in AD by the interaction of complement proteins with the aggregated forms of Aβ and tau (Rogers et al., 1992; Shen et al., 2001).

Notably, five genes, encoding the cysteine proteases Cathepsin B, Cathepsin L, Cathepsin S, and Cathepsin Z as well as the aspartyl protease Cathepsin D, were up-regulated in aged 5XFAD mice. Cathepsin D is a lysosomal enzyme found in neuritic plaques and is considered to be involved in APP processing (Schuur et al., 2011). Cataldo et al. (1995) showed an up-regulation of Cathepsin D mRNA in the pyramidal neurons of AD brains. The cysteine protease Cathepsin B has been proposed as an alternative candidate β-secretase in the regulated secretory pathway of neurons, where it produces Aβ by cleavage of the WT β-secretase site of APP (Hook et al., 2009; Wang et al., 2012). Hook et al. (2009) demonstrated that deletion of Cathepsin B in a hAPPwt transgenic mouse model significantly reduced the levels of Aβ40 and Aβ42. Therefore, Cathepsin B might be a valid target for developing inhibitors to lower brain Aβ levels in AD patients.

Another interesting gene that showed an up-regulated expression in aged 5XFAD mice is clusterin (*Clu*) also known as apolipoprotein J. Clusterin is a chaperone glycoprotein that affects many cellular processes, including inflammation. Clusterin is elevated in AD affected brain regions and CSF from AD patients (Lidström et al., 1998; Nilselid et al., 2006). Furthermore, it was found to be associated with AD in several large genome-wide association studies (GWAS) (Harold et al., 2009; Lambert et al., 2009; Carrasquillo et al., 2010). Recent studies suggest that Clusterin contributes to the pathology to AD through various pathways, including lipid metabolism, neuroinflammation, and apoptosis. Interestingly, it is reported to increase Aβ aggregation as well as Aβ clearance (Yu and Tan, 2012).

It should be noted that Inpp5d RNA was found to be differentially expressed in 5XFAD mice. This gene was recently described as a new locus for AD in a GWAS (Lambert et al., 2013). *Inpp5d* encodes a member of the inositol polyphosphate-5-phosphatase family of enzymes involved in second messenger signaling in myeloid cells. INPP5D influences pathways that are associated with cell proliferation and inflammatory responses (Medway and Morgan, 2014).

### Evidence for diverse molecular pathways

In addition to genes involved in inflammatory processes, DEGs in aged 5XFAD mice were also involved in cell communication and system development, signal transduction, synaptic transmission as well as regulation of gene expression and transcription.

We observed significant transcriptional changes of genes with synaptic function in aged 5XFAD mice. For instance, the gene products of *Bsn*, *Nrxn2*, *Kcnc1*, *Grm2*, and *Gng4* all play a role in synaptic transmission and are down-regulated in 12-month-old 5XFAD mice. *Syn1*, the gene encoding Synapsin1, a neuronal phosphoprotein associated with the cytoplasmic surface of synaptic vesicles, is significantly down-regulated in aged 5XFAD mice. It is involved in synapse formation and promotion of neurotransmitter release (Südhof, 1990; Jaffrey et al., 2002). Qin et al. (2004) showed that synapsin levels were also significantly decreased in the CA1 and the dentate gyrus in AD patients.

Wirz et al. (2013) studied the genome-wide gene expression of another AD double transgenic APP/PS1 mouse model using microarrays. A vast range of genes was altered in these APP/PS1_ΔEx9_ mice that are involved in immune response and inflammation. In contrast to our observations in 5XFAD mice, no changes in the expression of genes involved in synaptic plasticity or transmission were found. However, in AD patients dominant gene expression changes concerning synaptic plasticity or transmission were recently described in a genome-wide gene expression study of the prefrontal cortex (Bossers et al., 2010). It can be argued that deep sequencing and the use of 5XFAD mice are more informative and better suited to identify the expression changes in a model system of AD.

### Limitations of the study

Finally, it can be stated that RNA-Seq is a powerful technique to analyze the expression profiles in AD mice. The detection of hundreds of DEGs may offer a new perspective on the biological processes underlying the pathology of AD. However, even though there is a strong correlation between gene expression levels and abundances of the respectively corresponding proteins in mammalian cells (Lundberg et al., 2010), it has to be kept in mind that proteins, rather than mRNAs, are the main mediators of physiological processes and that there is a considerable body of data that suggests a major role for post-transcriptional processes in controlling protein abundances (Vogel and Marcotte, 2012).

While investigating the role of DEGs on the protein level is beyond the scope of this study, we believe that the presented dataset will provide an important source of information for the validation in both mouse and human tissue in independent studies. A wide range of detected genes were previously shown to be regulated in AD, however, a variety of DEGs in the studied mouse models were not previously associated with AD in humans. It remains to be seen if these genes are also regulated in AD cases.

In agreement with the German guidelines for animal care all animals were sacrificed by CO_2_ anesthesia. This treatment may lead to prefinal hypoxia in both the transgenic as well as in the control wildtype mice. However, it cannot be ruled out that hypoxia has distinct effects on transgenic mice inducing a different set of DEGs.

## Conclusion

In conclusion, we could (1) validate the Tg4–42 model expressing only Aβ_4–42_ as a valuable model for AD. The comparison with 5XFAD, an established plaque-developing AD mouse model, revealed a remarkable overlap in the molecular profile with the Tg4–42 model. Although the 5XFAD produces also Aβ_4–42_, Aβ_1–42_ is more abundant followed Aβ_1–40_ and pyroglutamated and non-pyroglutamated Aβ_3–42_. The jointly DEGs might indicate common pathways that are involved in the learning and memory decline apparent at 12 months of age in both transgenic models. (2) The pool of genes that showed differential expression exclusively in Tg4–42 is only associated to soluble Aβ_4–42_ as no extracellular plaques or other Aβ variants are found in this model. In addition, the robust CA1 neuron loss could also contribute to the differential expression profile. (3) As most of the genes with expression levels exclusively altered in 5XFAD mice belong to inflammation-associated pathways, we conclude that the majority is not associated with neuron loss and memory decline. (4) As expected, the deep sequencing approach identified a plethora of genes that have so far not been linked to AD, which might opens up new avenues of research into the etiology of this devastating neurodegenerative disorder.

## Author Contributions

Yvonne Bouter wrote the manuscript, contributed to experimental design, analyzed data and performed experiments. Tim Kacprowski analyzed data. Robert Weissmann performed experiments and analyzed data. Katharina Dietrich, Henning Borgers, Andreas Brauß, Christian Sperling and Lars R. Jensen performed experiments. Oliver Wirths, Mario Albrecht and Andreas W. Kuss contributed to experimental design. Thomas A. Bayer analyzed data and supervised experimental design and the entire project.

## Conflict of Interest Statement

The Tg4–42 mouse model is subject to a patent application by the University Medicine Goettingen.

## Footnotes

^1^http://hgdownload.cse.ucsc.edu/goldenPath/mm10/

^2^http://hgdownload.cse.ucsc.edu/goldenPath/mm10/database/refGene.txt.gz

^3^http://puma.princeton.edu/cgi-bin/source/sourceResult

^4^http://www.genecards.org

^5^http://severus.dbmi.pitt.edu/wiki-pi/index.php

^6^http://www.informatics.jax.org/

## References

[B1] Akiyama H. Barger S. Barnum S. Bradt B. Bauer J. Cole G. M. (2000). Inflammation and Alzheimer’s disease. Neurobiol. Aging 21, 383–421 10.1016/S0197-4580(00)00124-X 10858586 PMC3887148

[B2] Altar A. C. Vawter M. P. Ginsberg S. D. (2009). Target identification for CNS diseases by transcriptional profiling. Neuropsychopharmacology 34, 18–54 10.1038/npp.2008.172 18923405 PMC2675576

[B3] Alzheimer’s Association. (2012). 2012 Alzheimer’s disease facts and figures. Alzheimers Dement. 8, 131–168 10.1016/j.jalz.2012.02.001 22404854

[B4] Anders S. Huber W. (2010). Differential expression analysis for sequence count data. Genome Biol. 11, R106 10.1186/gb-2010-11-10-r106 20979621 PMC3218662

[B5] Arnaud L. Robakis N. K. Figueiredo-Pereira M. E. (2006). It may take inflammation, phosphorylation and ubiquitination to ‘tangle’ in Alzheimer’s disease. Neurodegener. Dis. 3, 313–319 10.1159/000095638 16954650

[B6] Ashburner M. Ball C. A. Blake J. A. Botstein D. Butler H. Cherry J. M. (2000). Gene ontology: tool for the unification of biology. The gene ontology consortium. Nat. Genet. 25, 25–29 10.1038/75556 10802651 PMC3037419

[B7] Bast T. Zhang W.-N. Feldon J. (2003). Dorsal hippocampus and classical fear conditioning to tone and context in rats: effects of local NMDA-receptor blockade and stimulation. Hippocampus 13, 657–675 10.1002/hipo.10115 12962312

[B8] Benilova I. Karran E. DeStrooper B. (2012). The toxic Aβ oligomer and Alzheimer’s disease: an emperor in need of clothes. Nat. Neurosci. 15, 349–357 10.1038/nn.3028 22286176

[B9] Bertram L. Tanzi R. E. (2001). Dancing in the dark? The status of late-onsetalzheimer’s disease genetics. J. Mol. Neurosci. 17, 127–136 10.1385/JMN:17:2:127 11816786

[B10] Blennow K. deLeon M. J. Zetterberg H. (2006). Alzheimer’s disease. Lancet 368, 387–403 10.1016/S0140-6736(06)69113-7 16876668

[B11] Bossers K. Wirz K. T. S. Meerhoff G. F. Essing A. H. W. van Dongen J. W. Houba P. (2010). Concerted changes in transcripts in the prefrontal cortex precede neuropathology in Alzheimer’s disease. Brain 133, 3699–3723 10.1093/brain/awq258 20889584

[B12] Bouter Y. Dietrich K. Wittnam J. L. Rezaei-Ghaleh N. Pillot T. Papot-Couturier S. (2013). N-truncated amyloid β (Aβ) 4-42 forms stable aggregates and induces acute and long-lasting behavioral deficits. Acta Neuropathol. 126, 189–205 10.1007/s00401-013-1129-2 23685882 PMC3722453

[B13] Breyhan H. Wirths O. Duan K. Marcello A. Rettig J. Bayer T. A. (2009). APP/PS1KI bigenic mice develop early synaptic deficits and hippocampus atrophy. Acta Neuropathol. 117, 677–685 10.1007/s00401-009-0539-7 19387667

[B14] Carrasquillo M. M. Belbin O. Hunter T. A. Ma L. Bisceglio G. D. Zou F. (2010). Replication of CLU, CR1, and PICALM associations with Alzheimer disease. Arch. Neurol. 67, 961–964 10.1001/archneurol.2010.147 20554627 PMC2919638

[B15] Carrette O. Demalte I. Scherl A. Yalkinoglu O. Corthals G. Burkhard P. (2003). A panel of cerebrospinal fluid potential biomarkers for the diagnosis of Alzheimer’s disease. Proteomics 3, 1486–1494 10.1002/pmic.200300470 12923774

[B16] Casas C. Sergeant N. Itier J.-M. Blanchard V. Wirths O. van der Kolk N. (2004). Massive CA1/2 neuronal loss with intraneuronal and N-terminal truncated Abeta42 accumulation in a novel Alzheimer transgenic model. Am. J. Pathol. 165, 1289–1300 10.1016/S0002-9440(10)63388-3 15466394 PMC1618627

[B17] Cataldo A. M. Barnett J. L. Berman S. A. Li J. Quarless S. Bursztajn S. (1995). Gene expression and cellular content of cathepsin D in Alzheimer’s disease brain: evidence for early up-regulation of the endosomal-lysosomal system. Neuron 14, 671–680 10.1016/0896-6273(95)90324-0 7695914

[B18] Chen G. Chen K. S. Knox J. Inglis J. Bernard A. Martin S. J. (2000). A learning deficit related to age and beta-amyloid plaques in a mouse model of Alzheimer’s disease. Nature 408, 975–979 10.1038/35050103 11140684

[B19] Cheng L. Quek C. Y. J. Sun X. Bellingham S. A. Hill A. F. (2013). The detection of microRNA associated with Alzheimer’s disease in biological fluids using next-generation sequencing technologies. Front. Genet. 4:150 10.3389/fgene.2013.00150 23964286 PMC3737441

[B20] Choi J. W. Kang S. M. Lee Y. Hong S. H. Sanek N. A. Young W. S. (2013). MicroRNA profiling in the mouse hypothalamus reveals oxytocin-regulating microRNA. J. Neurochem. 126, 331–337 10.1111/jnc.12308 23682839 PMC3716862

[B21] Christensen D. Z. Bayer T. A. Wirths O. (2010). Intracellular Aß triggers neuron loss in the cholinergic system of the APP/PS1KI mouse model of Alzheimer’s disease. Neurobiol. Aging 31, 1153–1163 10.1016/j.neurobiolaging.2008.07.022 18771817

[B22] Christensen D. Z. Kraus S. L. Flohr A. Cotel M.-C. Wirths O. Bayer T. A. (2008). Transient intraneuronal A beta rather than extracellular plaque pathology correlates with neuron loss in the frontal cortex of APP/PS1KI mice. Acta Neuropathol. 116, 647–655 10.1007/s00401-008-0451-6 18974993

[B23] Cocco C. D’Amato F. Noli B. Ledda A. Brancia C. Bongioanni P. (2010). Distribution of VGF peptides in the human cortex and their selective changes in Parkinson’s and Alzheimer’s diseases. J. Anat. 217, 683–693 10.1111/j.1469-7580.2010.01309.x 21039478 PMC3039181

[B24] Couillard-Despres S. Winner B. Schaubeck S. Aigner R. Vroemen M. Weidner N. (2005). Doublecortin expression levels in adult brain reflect neurogenesis. Eur. J. Neurosci. 21, 1–14 10.1111/j.1460-9568.2004.03813.x 15654838

[B25] Courtney E. Kornfeld S. Janitz K. Janitz M. (2010). Transcriptome profiling in neurodegenerative disease. J. Neurosci. Methods 193, 189–202 10.1016/j.jneumeth.2010.08.018 20800617

[B26] Cruz J. C. Tsai L.-H. (2004). Cdk5 deregulation in the pathogenesis of Alzheimer’s disease. Trends Mol. Med. 10, 452–458 10.1016/j.molmed.2004.07.001 15350898

[B27] Delacourte A. (1990). General and dramatic glial reaction in Alzheimer brains. Neurology 40, 33–37 10.1212/WNL.40.1.33 2296379

[B28] Dickey C. A. Loring J. F. Montgomery J. Gordon M. N. Eastman P. S. Morgan D. (2003). Selectively reduced expression of synaptic plasticity-related genes in amyloid precursor protein + presenilin-1 transgenic mice. J. Neurosci. 23, 5219–5226 12832546 10.1523/JNEUROSCI.23-12-05219.2003 PMC6741153

[B29] España J. Giménez-Llort L. Valero J. Miñano A. Rábano A. Rodriguez-Alvarez J. (2010). Intraneuronal beta-amyloid accumulation in the amygdala enhances fear and anxiety in Alzheimer’s disease transgenic mice. Biol. Psychiatry 67, 513–521 10.1016/j.biopsych.2009.06.015 19664757

[B30] Farris W. Mansourian S. Chang Y. Lindsley L. Eckman E. A. Frosch M. P. (2003). Insulin-degrading enzyme regulates the levels of insulin, amyloid beta-protein, and the beta-amyloid precursor protein intracellular domain in vivo. Proc. Natl. Acad. Sci. U.S.A. 100, 4162–4167 10.1073/pnas.0230450100 12634421 PMC153065

[B31] George A. J. Gordon L. Beissbarth T. Koukoulas I. Holsinger R. M. Perreau V. (2010). A serial analysis of gene expression profile of the Alzheimer’s disease Tg2576 mouse model. Neurotox. Res. 17, 360–379 10.1007/s12640-009-9112-3 19760337

[B32] Glaser S. Schaft J. Lubitz S. Vintersten K. van der Hoeven F. Tufteland K. R. (2006). Multiple epigenetic maintenance factors implicated by the loss of Mll2 in mouse development. Development 133, 1423–1432 10.1242/dev.02302 16540515

[B33] Haass C. Selkoe D. J. (2007). Soluble protein oligomers in neurodegeneration: lessons from the Alzheimer’s amyloid beta-peptide. Nat. Rev. Mol. Cell Biol. 8, 101–112 10.1038/nrm2101 17245412

[B34] Hardy J. Allsop D. (1991). Amyloid deposition as the central event in the aetiology of Alzheimer’s disease. Trends Pharmacol. Sci. 12, 383–388 10.1016/0165-6147(91)90609-V 1763432

[B35] Harold D. Abraham R. Hollingworth P. Sims R. Gerrish A. Hamshere M. L. (2009). Genome-wide association study identifies variants at CLU and PICALM associated with Alzheimer’s disease. Nat. Genet. 41, 1088–1093 10.1038/ng.440 19734902 PMC2845877

[B36] Haughey N. J. Liu D. Nath A. Borchard A. C. Mattson M. P. (2002a). Disruption of neurogenesis in the subventricular zone of adult mice, and in human cortical neuronal precursor cells in culture, by amyloid beta-peptide: implications for the pathogenesis of Alzheimer’s disease. Neuromolecular Med. 1, 125–135 10.1385/NMM:1:2:125 12025858

[B37] Haughey N. J. Nath A. Chan S. L. Borchard A. C. Rao M. S. Mattson M. P. (2002b). Disruption of neurogenesis by amyloid beta-peptide, and perturbed neural progenitor cell homeostasis, in models of Alzheimer’s disease. J. Neurochem. 83, 1509–1524 10.1046/j.1471-4159.2002.01267.x 12472904

[B38] Haupt C. Leppert J. Rönicke R. Meinhardt J. Yadav J. K. Ramachandran R. (2012). Structural basis of β-amyloid-dependent synaptic dysfunctions. Angew. Chem. Int. Ed. Engl. 51, 1576–1579 10.1002/anie.201105638 22234970

[B39] Hillmann A. Hahn S. Schilling S. Hoffmann T. Demuth H.-U. Bulic B. (2012). No improvement after chronic ibuprofen treatment in the 5XFAD mouse model of Alzheimer’s disease. Neurobiol. Aging 33, .e39–.e50 10.1016/j.neurobiolaging.2011.08.006 21943956

[B40] Hirokawa N. Niwa S. Tanaka Y. (2010). Molecular motors in neurons: transport mechanisms and roles in brain function, development, and disease. Neuron 68, 610–638 10.1016/j.neuron.2010.09.039 21092854

[B41] Hirokawa N. Noda Y. Tanaka Y. Niwa S. (2009). Kinesin superfamily motor proteins and intracellular transport. Nat. Rev. Mol. Cell Biol. 10, 682–696 10.1038/nrm2774 19773780

[B42] Hook V. Y. Kindy M. Reinheckel T. Peters C. Hook G. (2009). Genetic cathepsin B deficiency reduces beta-amyloid in transgenic mice expressing human wild-type amyloid precursor protein. Biochem. Biophys. Res. Commun. 386, 284–288 10.1016/j.bbrc.2009.05.131 19501042 PMC2753505

[B43] Jaffrey S. R. Benfenati F. Snowman A. M. Czernik A. J. Snyder S. H. (2002). Neuronal nitric-oxide synthase localization mediated by a ternary complex with synapsin and CAPON. Proc. Natl. Acad. Sci. U.S.A. 99, 3199–3204 10.1073/pnas.261705799 11867766 PMC122496

[B44] Jahn H. Wittke S. Zürbig P. Raedler T. J. Arlt S. Kellmann M. (2011). Peptide fingerprinting of Alzheimer’s disease in cerebrospinal fluid: identification and prospective evaluation of new synaptic biomarkers. PLoS ONE 6:e26540 10.1371/journal.pone.0026540 22046305 PMC3202544

[B45] Jawhar S. Trawicka A. Jenneckens C. Bayer T. A. Wirths O. (2010). Motor deficits, neuron loss, and reduced anxiety coinciding with axonal degeneration and intraneuronal Abeta aggregation in the 5XFAD mouse model of Alzheimer’s disease. Neurobiol. Aging 33, .e29–.e40 10.1016/j.neurobiolaging.2010.05.027 20619937

[B46] Jawhar S. Wirths O. Bayer T. A. (2011). Pyroglutamate amyloid-β (Aβ): a hatchet man in Alzheimer disease. J. Biol. Chem. 286, 38825–38832 10.1074/jbc.R111.288308 21965666 PMC3234707

[B47] Jin K. Peel A. L. Mao X. O. Xie L. Cottrell B. A. Henshall D. C. (2004). Increased hippocampal neurogenesis in Alzheimer’s disease. Proc. Natl. Acad. Sci. U.S.A. 101, 343–347 10.1073/pnas.2634794100 14660786 PMC314187

[B48] Kalinin S. Richardson J. C. Feinstein D. L. (2009). A PPARdelta agonist reduces amyloid burden and brain inflammation in a transgenic mouse model of Alzheimer’s disease. Curr. Alzheimer Res. 6, 431–437 10.2174/156720509789207949 19874267

[B49] Kanekiyo T. Cirrito J. R. Liu C.-C. Shinohara M. Li J. Schuler D. R. (2013). Neuronal clearance of amyloid-β by endocytic receptor LRP1. J. Neurosci. 33, 19276–19283 10.1523/JNEUROSCI.3487-13.2013 24305823 PMC3850043

[B50] Kerimoglu C. Agis-Balboa R. C. Kranz A. Stilling R. Bahari-Javan S. Benito-Garagorri E. (2013). Histone-methyltransferase MLL2 (KMT2B) is required for memory formation in mice. J. Neurosci. 33, 3452–3464 10.1523/JNEUROSCI.3356-12.2013 23426673 PMC6619533

[B51] Kishimoto Y. Higashihara E. Fukuta A. Nagao A. Kirino Y. (2013). Early impairment in a water-finding test in a longitudinal study of the Tg2576 mouse model of Alzheimer’s disease. Brain Res. 1491, 117–126 10.1016/j.brainres.2012.10.066 23142630

[B52] Klein W. L. (2002). Abeta toxicity in Alzheimer’s disease: globular oligomers (ADDLs) as new vaccine and drug targets. Neurochem. Int. 41, 345–352 10.1016/S0197-0186(02)00050-5 12176077

[B53] Ko H. S. Uehara T. Tsuruma K. Nomura Y. (2004). Ubiquilin interacts with ubiquitylated proteins and proteasome through its ubiquitin-associated and ubiquitin-like domains. FEBS Lett. 566, 110–114 10.1016/j.febslet.2004.04.031 15147878

[B54] Kondo M. Takei Y. Hirokawa N. (2012). Motor protein KIF1A is essential for hippocampal synaptogenesis and learning enhancement in an enriched environment. Neuron 73, 743–757 10.1016/j.neuron.2011.12.020 22365548

[B55] Lambert J.-C. Heath S. Even G. Campion D. Sleegers K. Hiltunen M. (2009). Genome-wide association study identifies variants at CLU and CR1 associated with Alzheimer’s disease. Nat. Genet. 41, 1094–1099 10.1038/ng.439 19734903

[B56] Lambert J.-C. Ibrahim-Verbaas C. A. Harold D. Naj A. C. Sims R. Bellenguez C. (2013). Meta-analysis of 74,046 individuals identifies 11 new susceptibility loci for Alzheimer’s disease. Nat. Genet. 45, 1452–1458 10.1038/ng.2802 24162737 PMC3896259

[B57] Leskovjan A. C. Kretlow A. Lanzirotti A. Barrea R. Vogt S. Miller L. M. (2011). Increased brain iron coincides with early plaque formation in a mouse model of Alzheimer’s disease. Neuroimage 55, 32–38 10.1016/j.neuroimage.2010.11.073 21126592 PMC3031776

[B58] Lesné S. E. Sherman M. A. Grant M. Kuskowski M. Schneider J. A. Bennett D. A. (2013). Brain amyloid-β oligomers in ageing and Alzheimer’s disease. Brain 136, 1383–1398 10.1093/brain/awt062 23576130 PMC3634198

[B59] Li X. Buxbaum J. N. (2011). Transthyretin and the brain re-visited: is neuronal synthesis of transthyretin protective in Alzheimer’s disease? Mol. Neurodegener. 6, 79 10.1186/1750-1326-6-79 22112803 PMC3267701

[B60] Lidström A. M. Bogdanovic N. Hesse C. Volkman I. Davidsson P. Blennow K. (1998). Clusterin (apolipoprotein J) protein levels are increased in hippocampus and in frontal cortex in Alzheimer’s disease. Exp. Neurol. 154, 511–521 10.1006/exnr.1998.6892 9878186

[B61] Lovell M. A. Robertson J. D. Teesdale W. J. Campbell J. L. Markesbery W. R. (1998). Copper, iron and zinc in Alzheimer’s disease senile plaques. J. Neurol. Sci. 158, 47–52 10.1016/S0022-510X(98)00092-6 9667777

[B62] Lundberg E. Fagerberg L. Klevebring D. Matic I. Geiger T. Cox J. (2010). Defining the transcriptome and proteome in three functionally different human cell lines. Mol. Syst. Biol. 6, 450 10.1038/msb.2010.106 21179022 PMC3018165

[B63] Masters C. L. Simms G. Weinman N. A. Multhaup G. McDonald B. L. Beyreuther K. (1985). Amyloid plaque core protein in Alzheimer disease and Down syndrome. Proc. Natl. Acad. Sci. U.S.A. 82, 4245–4249 10.1073/pnas.82.12.4245 3159021 PMC397973

[B64] McLean C. A. Cherny R. A. Fraser F. W. Fuller S. J. Smith M. J. Beyreuther K. (1999). Soluble pool of Abeta amyloid as a determinant of severity of neurodegeneration in Alzheimer’s disease. Ann. Neurol. 46, 860–866 10.1002/1531-8249(199912)46:6<860::AID-ANA8>3.0.CO;2-M 10589538

[B65] Medway C. Morgan K. (2014). Review: the genetics of Alzheimer’s disease; putting flesh on the bones. Neuropathol. Appl. Neurobiol. 40, 97–105 10.1111/nan.12101 24443964 PMC4282344

[B66] Miller D. L. Papayannopoulos I. A. Styles J. Bobin S. A. Lin Y. Y. Biemann K. (1993). Peptide compositions of the cerebrovascular and senile plaque core amyloid deposits of Alzheimer’s disease. Arch. Biochem. Biophys. 301, 41–52 10.1006/abbi.1993.1112 8442665

[B67] Miners J. S. Baig S. Tayler H. Kehoe P. G. Love S. (2009). Neprilysin and insulin-degrading enzyme levels are increased in Alzheimer disease in relation to disease severity. J. Neuropathol. Exp. Neurol. 68, 902–914 10.1097/NEN.0b013e3181afe475 19606063

[B68] Moechars D. Dewachter I. Lorent K. Reversé D. Baekelandt V. Naidu A. (1999). Early phenotypic changes in transgenic mice that overexpress different mutants of amyloid precursor protein in brain. J. Biol. Chem. 274, 6483–6492 10.1074/jbc.274.10.6483 10037741

[B69] Moechars D. Lorent K. Strooper B. Dewachter I. van Leuven F. (1996). Expression in brain of amyloid precursor protein mutated in the alpha-secretase site causes disturbed behavior, neuronal degeneration and premature death in transgenic mice. EMBO J. 15, 1265–1274 8635459 PMC450029

[B70] Morris R. (1984). Developments of a water-maze procedure for studying spatial learning in the rat. J. Neurosci. Methods 11, 47–60 10.1016/0165-0270(84)90007-4 6471907

[B71] Nagalakshmi U. Wang Z. Waern K. Shou C. Raha D. Gerstein M. (2008). The transcriptional landscape of the yeast genome defined by RNA sequencing. Science 320, 1344–1349 10.1126/science.1158441 18451266 PMC2951732

[B72] Näslund J. Haroutunian V. Mohs R. Davis K. L. Davies P. Greengard P. (2000). Correlation between elevated levels of amyloid beta-peptide in the brain and cognitive decline. JAMA 283, 1571–1577 10.1001/jama.283.12.1571 10735393

[B73] Nikolic M. Dudek H. Kwon Y. T. Ramos Y. F. Tsai L. H. (1996). The cdk5/p35 kinase is essential for neurite outgrowth during neuronal differentiation. Genes Dev. 10, 816–825 10.1101/gad.10.7.816 8846918

[B74] Nilselid A.-M. Davidsson P. Nägga K. Andreasen N. Fredman P. Blennow K. (2006). Clusterin in cerebrospinal fluid: analysis of carbohydrates and quantification of native and glycosylated forms. Neurochem. Int. 48, 718–728 10.1016/j.neuint.2005.12.005 16490286

[B75] Oakley H. Cole S. L. Logan S. Maus E. Shao P. Craft J. (2006). Intraneuronal beta-amyloid aggregates, neurodegeneration, and neuron loss in transgenic mice with five familial Alzheimer’s disease mutations: potential factors in amyloid plaque formation. J. Neurosci. 26, 10129–10140 10.1523/JNEUROSCI.1202-06.2006 17021169 PMC6674618

[B76] Ohno M. (2009). Failures to reconsolidate memory in a mouse model of Alzheimer’s disease. Neurobiol. Learn. Mem. 92, 455–459 10.1016/j.nlm.2009.05.001 19435612 PMC2772829

[B77] Pfaffl M. W. Horgan G. W. Dempfle L. (2002). Relative expression software tool (REST) for group-wise comparison and statistical analysis of relative expression results in real-time PCR. Nucleic Acids Res. 30, e36 10.1093/nar/30.9.e36 11972351 PMC113859

[B78] Pham E. Crews L. Ubhi K. Hansen L. Adame A. Cartier A. (2010). Progressive accumulation of amyloid-beta oligomers in Alzheimer’s disease and in amyloid precursor protein transgenic mice is accompanied by selective alterations in synaptic scaffold proteins. FEBS J. 277, 3051–3067 10.1111/j.1742-4658.2010.07719.x 20573181 PMC2933033

[B79] Phillips R. G. LeDoux J. E. (1992). Differential contribution of amygdala and hippocampus to cued and contextual fear conditioning. Behav. Neurosci. 106, 274–285 10.1037/0735-7044.106.2.274 1590953

[B80] Portelius E. Bogdanovic N. Gustavsson M. K. Volkmann I. Brinkmalm G. Zetterberg H. (2010). Mass spectrometric characterization of brain amyloid beta isoform signatures in familial and sporadic Alzheimer’s disease. Acta Neuropathol. 120, 185–193 10.1007/s00401-010-0690-1 20419305 PMC3568930

[B81] Prelli F. Castaño E. Glenner G. G. Frangione B. (1988). Differences between vascular and plaque core amyloid in Alzheimer’s disease. J. Neurochem. 51, 648–651 10.1111/j.1471-4159.1988.tb01087.x 3292706

[B82] Price J. L. Morris J. C. (1999). Tangles and plaques in nondemented aging and “preclinical” Alzheimer’s disease. Ann. Neurol. 45, 358–368 10.1002/1531-8249(199903)45:3<358::AID-ANA12>3.0.CO;2-X 10072051

[B83] Qin S. Hu X.-Y. Xu H. Zhou J.-N. (2004). Regional alteration of synapsin I in the hippocampal formation of Alzheimer’s disease patients. Acta Neuropathol. 107, 209–215 10.1007/s00401-003-0800-4 14673601

[B84] Rademakers R. Sleegers K. Theuns J. van den Broeck M. Bel Kacem S. Nilsson L.-G. (2005). Association of cyclin-dependent kinase 5 and neuronal activators p35 and p39 complex in early-onsetalzheimer’s disease. Neurobiol. Aging 26, 1145–1151 10.1016/j.neurobiolaging.2004.10.003 15917097

[B85] Risso D. Schwartz K. Sherlock G. Dudoit S. (2011). GC-content normalization for RNA-Seq data. BMC Bioinformatics 12:480 10.1186/1471-2105-12-480 22177264 PMC3315510

[B86] Roberts B. R. Ryan T. M. Bush A. I. Masters C. L. Duce J. A. (2012). The role of metallobiology and amyloid-β peptides in Alzheimer’s disease. J. Neurochem. 120(Suppl. 1), 149–166 10.1111/j.1471-4159.2011.07500.x 22121980

[B87] Rogers J. Cooper N. R. Webster S. Schultz J. McGeer P. L. Styren S. D. (1992). Complement activation by beta-amyloid in Alzheimer disease. Proc. Natl. Acad. Sci. U.S.A. 89, 10016–10020 10.1073/pnas.89.21.10016 1438191 PMC50268

[B88] Roychaudhuri R. Yang M. Hoshi M. M. Teplow D. B. (2009). Amyloid beta-protein assembly and Alzheimer disease. J. Biol. Chem. 284, 4749–4753 10.1074/jbc.R800036200 18845536 PMC3837440

[B89] Schmitz C. Rutten B. P. F. Pielen A. Schäfer S. Wirths O. Tremp G. (2004). Hippocampal neuron loss exceeds amyloid plaque load in a transgenic mouse model of Alzheimer’s disease. Am. J. Pathol. 164, 1495–1502 10.1016/S0002-9440(10)63235-X 15039236 PMC1615337

[B90] Schuur M. Ikram M. A. van Swieten J. C. Isaacs A. Vergeer-Drop J. M. Hofman A. (2011). Cathepsin D gene and the risk of Alzheimer’s disease: a population-based study and meta-analysis. Neurobiol. Aging 32, 1607–1614 10.1016/j.neurobiolaging.2009.10.011 19926167

[B91] Selkoe D. J. (1998). The cell biology of beta-amyloid precursor protein and presenilin in Alzheimer’s disease. Trends Cell Biol. 8, 447–453 10.1016/S0962-8924(98)01363-4 9854312

[B92] Selkoe D. J. (2011). Resolving controversies on the path to Alzheimer’s therapeutics. Nat. Med. 17, 1060–1065 10.1038/nm.2460 21900936

[B93] Selwood S. P. Parvathy S. Cordell B. Ryan H. S. Oshidari F. Vincent V. (2009). Gene expression profile of the PDAPP mouse model for Alzheimer’s disease with and without apolipoprotein E. Neurobiol. Aging 30, 574–590 10.1016/j.neurobiolaging.2007.08.006 17904698

[B94] Shen Y. Lue L. Yang L. Roher A. Kuo Y. Strohmeyer R. (2001). Complement activation by neurofibrillary tangles in Alzheimer’s disease. Neurosci. Lett. 305, 165–168 10.1016/S0304-3940(01)01842-0 11403931

[B95] Shukla V. Skuntz S. Pant H. C. (2012). Deregulated Cdk5 activity is involved in inducing Alzheimer’s disease. Arch. Med. Res. 43, 655–662 10.1016/j.arcmed.2012.10.015 23142263 PMC3532552

[B96] Snyder S. E. Salton S. R. (1998). Expression of VGF mRNA in the adult rat central nervous system. J. Comp. Neurol. 394, 91–105 10.1002/(SICI)1096-9861(19980427)394:1<91::AID-CNE7>3.3.CO;2-B 9550144

[B97] Stein T. D. Johnson J. A. (2002). Lack of neurodegeneration in transgenic mice overexpressing mutant amyloid precursor protein is associated with increased levels of transthyretin and the activation of cell survival pathways. J. Neurosci. 22, 7380–7388 12196559 10.1523/JNEUROSCI.22-17-07380.2002 PMC6758007

[B98] Stover K. R. Brown R. E. (2012). Age-related changes in visual acuity, learning and memory in the APPswe/PS1dE9 mouse model of Alzheimer’s disease. Behav. Brain Res. 231, 75–85 10.1016/j.bbr.2012.02.044 22409975

[B99] Südhof T. C. (1990). The structure of the human synapsin I gene and protein. J. Biol. Chem. 265, 7849–7852 2110562

[B100] Sultan M. Schulz M. H. Richard H. Magen A. Klingenhoff A. Scherf M. (2008). A global view of gene activity and alternative splicing by deep sequencing of the human transcriptome. Science 321, 956–960 10.1126/science.1160342 18599741

[B101] Sutherland G. T. Janitz M. Kril J. J. (2011). Understanding the pathogenesis of Alzheimer’s disease: will RNA-Seq realize the promise of transcriptomics? J. Neurochem. 116, 937–946 10.1111/j.1471-4159.2010.07157.x 21175619

[B102] Takemura R. Nakata T. Okada Y. Yamazaki H. Zhang Z. Hirokawa N. (1996). mRNA expression of KIF1A, KIF1B, KIF2, KIF3A, KIF3B, KIF4, KIF5, and cytoplasmic dynein during axonal regeneration. J. Neurosci. 16, 31–35 8613797 10.1523/JNEUROSCI.16-01-00031.1996 PMC6578719

[B103] Tan T. C. Valova V. A. Malladi C. S. Graham M. E. Berven L. A. Jupp O. J. (2003). Cdk5 is essential for synaptic vesicle endocytosis. Nat. Cell Biol. 5, 701–710 10.1038/ncb1020 12855954

[B104] Thakker-Varia S. Alder J. (2009). Neuropeptides in depression: role of VGF. Behav. Brain Res. 197, 262–278 10.1016/j.bbr.2008.10.006 18983874 PMC2648305

[B105] Twine N. A. Janitz K. Wilkins M. R. Janitz M. (2011). Whole transcriptome sequencing reveals gene expression and splicing differences in brain regions affected by Alzheimer’s disease. PLoS ONE 6:e16266 10.1371/journal.pone.0016266 21283692 PMC3025006

[B106] Upadhaya R. A. Scheibe F. Kosterin I. Abramowski D. Gerth J. Kumar S. (2013). The type of Aβ-related neuronal degeneration differs between amyloid precursor protein (APP23) and amyloid β-peptide (APP48) transgenic mice. Acta Neuropathol. Commun. 1, 77 10.1186/2051-5960-1-77 24252227 PMC4046770

[B107] van Bakel H. Nislow C. Blencowe B. J. Hughes T. R. (2010). Most “dark matter” transcripts are associated with known genes. PLoS Biol. 8:e1000371 10.1371/journal.pbio.1000371 20502517 PMC2872640

[B108] van den Pol A. N. Bina K. Decavel C. Ghosh P. (1994). VGF expression in the brain. J. Comp. Neurol. 347, 455–469 10.1002/cne.903470311 7822494

[B109] Verwer R. W. H. Sluiter A. A. Balesar R. A. Baayen J. C. Noske D. P. Dirven C. M. F. (2007). Mature astrocytes in the adult human neocortex express the early neuronal marker doublecortin. Brain 130, 3321–3335 10.1093/brain/awm264 18055496

[B110] Vogel C. Marcotte E. M. (2012). Insights into the regulation of protein abundance from proteomic and transcriptomic analyses. Nat. Rev. Genet. 13, 227–232 10.1038/nrg3185 22411467 PMC3654667

[B111] Wang C. Sun B. Zhou Y. Grubb A. Gan L. (2012). Cathepsin B degrades amyloid-β in mice expressing wild-type human amyloid precursor protein. J. Biol. Chem. 287, 39834–39841 10.1074/jbc.M112.371641 23024364 PMC3501032

[B112] Wang Z. Gerstein M. Snyder M. (2009). RNA-Seq: a revolutionary tool for transcriptomics. Nat. Rev. Genet. 10, 57–63 10.1038/nrg2484 19015660 PMC2949280

[B113] Watt N. T. Whitehouse I. J. Hooper N. M. (2010). The role of zinc in Alzheimer’s disease. Int. J. Alzheimers Dis. 2011, 971021 10.4061/2011/971021 21197404 PMC3010690

[B114] Wirths O. Bayer T. A. (2012). Intraneuronal Aβ accumulation and neurodegeneration: lessons from transgenic models. Life Sci. 91, 1148–1152 10.1016/j.lfs.2012.02.001 22401905

[B115] Wirths O. Multhaup G. Bayer T. A. (2004). A modified beta-amyloid hypothesis: intraneuronal accumulation of the beta-amyloid peptide – the first step of a fatal cascade. J. Neurochem. 91, 513–520 10.1111/j.1471-4159.2004.02737.x 15485483

[B116] Wirz K. T. S. Bossers K. Stargardt A. Kamphuis W. Swaab D. F. Hol E. M. (2013). Cortical beta amyloid protein triggers an immune response, but no synaptic changes in the APPswe/PS1dE9 Alzheimer’s disease mouse model. Neurobiol. Aging 34, 1328–1342 10.1016/j.neurobiolaging.2012.11.008 23245294

[B117] Wittnam J. L. Portelius E. Zetterberg H. Gustavsson M. K. Schilling S. Koch B. (2012). Pyroglutamate amyloid β (Aβ) aggravates behavioral deficits in transgenic amyloid mouse model for Alzheimer disease. J. Biol. Chem. 287, 8154–8162 10.1074/jbc.M111.308601 22267726 PMC3318696

[B118] Wu Z.-L. Ciallella J. R. Flood D. G. O’Kane T. M. Bozyczko-Coyne D. Savage M. J. (2006). Comparative analysis of cortical gene expression in mouse models of Alzheimer’s disease. Neurobiol. Aging 27, 377–386 10.1016/j.neurobiolaging.2005.02.010 15927307

[B119] Yahata N. Asai M. Kitaoka S. Takahashi K. Asaka I. Hioki H. (2011). Anti-Aβ drug screening platform using human iPS cell-derived neurons for the treatment of Alzheimer’s disease. PLoS ONE 6:e25788 10.1371/journal.pone.0025788 21984949 PMC3184175

[B120] Yu J.-T. Tan L. (2012). The role of clusterin in Alzheimer’s disease: pathways, pathogenesis, and therapy. Mol. Neurobiol. 45, 314–326 10.1007/s12035-012-8237-1 22274961

